# Deletion of the Syncytin A receptor *Ly6e* impairs syncytiotrophoblast fusion and placental morphogenesis causing embryonic lethality in mice

**DOI:** 10.1038/s41598-018-22040-2

**Published:** 2018-03-02

**Authors:** Michael B. Langford, Jennifer E. Outhwaite, Martha Hughes, David R. C. Natale, David G. Simmons

**Affiliations:** 1 School of Biomedical Sciences, Faculty of Medicine and Biomedical Sciences, The University of Queensland, St. Lucia, Queensland, Australia; 2 Department of Comparative Biology and Experimental Medicine, Faculty of Veterinary Medicine, University of Calgary, Calgary, AB Canada; 3 Department of Reproductive Medicine, School of Medicine, University of California, San Diego, California, USA; 4 Present Address: Queensland Brain Institute, Faculty of Medicine and Biomedical Sciences, The University of Queensland, St. Lucia Queensland, Australia

## Abstract

Fetal growth and survival is dependent on the elaboration and propinquity of the fetal and maternal circulations within the placenta. Central to this is the formation of the interhaemal membrane, a multi-cellular lamina facilitating exchange of oxygen, nutrients and metabolic waste products between the mother and fetus. In rodents, this cellular barrier contains two transporting layers of syncytiotrophoblast, which are multinucleated cells that form by cell-cell fusion. Previously, we reported the expression of the GPI-linked cell surface protein LY6E by the syncytial layer closest to the maternal sinusoids of the mouse placenta (syncytiotrophoblast layer I). LY6E has since been shown to be a putative receptor for the fusogenic protein responsible for fusion of syncytiotrophoblast layer I, Syncytin A. In this report, we demonstrate that LY6E is essential for the normal fusion of syncytiotrophoblast layer I, and for the proper morphogenesis of both fetal and maternal vasculatures within the placenta. Furthermore, specific inactivation of *Ly6e* in the epiblast, but not in placenta, is compatible with embryonic development, indicating the embryonic lethality reported for *Ly6e*^−/−^ embryos is most likely placental in origin.

## Introduction

The rodent chorioallantoic placenta develops an extensive network of fetal capillaries and maternal blood sinusoids to provide the substantial surface area required for the transfer of nutrients to the fetus during pregnancy. Layers of trophoblast cells form a thin, specialised interface called the interhaemal membrane that mediates selective and bidirectional exchange between the fetal and maternal blood compartments. While the type and number of trophoblast cell layers comprising the interhaemal membrane varies among mammalian species^1^, a common feature in many, including the haemochorial placentae of both mice and humans, is the syncytial nature of the transporting trophoblast cell layer(s). In mice, the fetal capillaries and maternal sinusoids are tortuously intertwined within the innermost layer of the placenta called the “labyrinth”, and the interhaemal membrane that separates the blood compartments is composed of three layers of trophoblast cells; one fenestrated mononuclear trophoblast giant cell layer lining the maternal sinusoids (S-TGCs) and two tightly apposed syncytial trophoblast layers (SynT-I and SynT-II) which lay adjacent to the endothelial cells of the fetal capillaries (Fig. S1). Patterning for this ordered cellular lamina is laid down in the chorion early in development, prior to morphogenesis of the labyrinth^2^.

Labyrinth formation ostensibly begins ~embryonic day (E)8-E8.5 when the allantois, containing progenitors of the fetal vasculature, attaches to, and spreads across, the surface of the chorion. At the time of chorioallantoic attachment, the chorion is a flat disc of trophoblast progenitor cells situated beneath the developing maternal blood sinuses^3^. However, the chorionic disc is not homogeneous; clusters of cells residing along the leading edge express the transcription factor *Gcm1*, and mark sites where folding is initiated to create simple rudimentary branches^4–7^. Allantoic tissue with its associated blood vessels grows into the space vacated by the chorionic folding, while *Gcm1*^+^ trophoblast cells at the tips of the primary branches elongate, differentiate and fuse to form the inner most layer of syncytia (Syncytiotrophoblast layer-II, or SynT-II cells)^2,8,9^. These early primary branches continue to extend deeper into the chorionic plate bringing SynT-II cells into contact with cells on the distal side of the chorion, which express the fusogenic gene *Syna*^2^. Electron microscopy studies suggested that contact between *Gcm1*^+^ SynT-II cells and the more distal *Syna*^+^ cells is the trigger for the lateral fusion of *Syna*^+^ cells into the first layer of syncytia (SynT-I cells)^8^; more recent cellular ablation experiments have corroborated this notion, as SynT-I fusion is inhibited in the absence of SynT-II cells^10^. SynT-I and SynT-II cells become intimately juxtaposed and ultimately function together as the transporting epithelium of the definitive placenta. Continued elongation of the primary branches extends them into the developing maternal sinuses at the base of the ectoplacental cone, which are lined by *Hand1*^+^ cells that are the likely progenitors of the mononuclear sinusoidal trophoblast giant cells (S-TGCs)^2^. Thus, a model has emerged: as the primary branch points, backfilled and vascularised by allantoic tissue, traverse the chorion they successively accumulate each trophoblast layer comprising the interhaemal membrane of the definitive placenta. Further branching morphogenesis elaborates these rudimentary branches into a complex structure to produce the immense surface area for fetal-maternal exchange. Disturbances in the development and elaboration of this architecture, or in the cellular structure and integrity of the interhaemal membrane, result in impaired placental function and adverse developmental outcomes for the fetus such as fetal growth restriction, or in severe cases mid-gestation lethality^11,12^.

Significant detail regarding the specification and fusion of SynT-II cells is now emerging. The transcription factor *Gcm1* plays a central role in specifying SynT-II cell identity and orchestrating cell-cell fusion; the initiation of branching morphogenesis and the fusion of chorionic trophoblast into syncytiotrophoblast cells is blocked in *Gcm1* knockout mice^6,7^. GCM1 activity choreographs the fusion of chorionic trophoblast cells into SynT-II by inducing expression of *Synb*^2,13–15^, a fusogenic endogenous retroviral gene essential for SynT-II fusion^15^. Other signalling pathways have also been shown to regulate SynT-II fusion, such as those activated by the proprotein convertase furin^16^ or vasohibin-2 (*Vash2*)^17^, although it is not currently known if these pathways are downstream of *Gcm1* or represent parallel regulatory pathways. Furthermore, ERK/MAPK signalling is instrumental in regulating syncytiotrophoblast fusion in mice^9,10^. Blocking SynT-II fusion downstream of *Gcm1* impairs placental function and causes fetal growth restriction, but is not itself embryonic lethal^15,17^, as an upregulation of gap junctions appears able to compensate for the loss of fusion between SynT-II cells^15^.

In contrast to SynT-II cells, comparatively little is known about the specification and formation of the first layer of syncytiotrophoblast (SynT-I cells). What is known is that cells along the distal side of the E8.5 chorion, just underlying the developing maternal sinusoids within the EPC, begin to express the fusogenic endogenous retroviral gene *Syna*, and ultimately fuse laterally to become SynT-I cells^2,18^. Genetic deletion of *Syna* prevents SynT-I fusion and results in complete embryonic lethality by E14.5^19^. However, the transcription factors and signalling mechanisms responsible for specifying these cells and driving *Syna* expression remain unknown. We previously identified the membrane protein LY6E as being expressed by SynT-I cells early in development, and found that isolated *Ly6e*^+^ trophoblast stem cells formed multinucleated cells *in vitro*, suggesting this protein may play some role in SynT-I formation and function^20^. While identified in 2005, the receptors for both Syncytin A and Syncytin B have not been reported, until very recently, when Bacquin A, *et al*. showed that LY6E can function as a receptor for Syncytin A^21^.

Deletion of *Ly6e*, a member of the Ly6 superfamily^22–26^ and also known as stem cell antigen 2 (SCA2), retinoic acid induced gene E (RIGE), and thymic shared antigen 1 (TSA1), results in embryonic lethality at mid-gestation (E15.5), reportedly due to dilated cardiomyopathy and heart failure^27^. Interestingly, *Ly6e* expression in the developing heart itself is negligible, making this a perplexing phenotype. However, developmental heart defects have been previously observed as secondary phenotypes downstream of primary placental phenotypes^28,29^, suggesting a possible developmental “placenta-heart axis”. As *Ly6e* is expressed in SynT-I cells of the placenta^20^ and defects in this layer result in mid-gestational embryonic lethality^19^, we hypothesised that placental defects may in fact underlie the embryonic lethality seen in *Ly6e* mutants. In the current study, we examined placental development in *Ly6e*^−/−^ embryos and found defects in both the morphogenesis of the labyrinth architecture as well as impaired cell-cell fusion in the SynT-I layer. Furthermore, we found evidence of disruption to cell layers adjacent to SynT-I cells within the interhaemal membrane, highlighting the interdependency of the cell layers in the formation and maintenance of the interhaemal membrane. Importantly, these placental defects precede the heart defects and embryonic lethality that have been previously described in *Ly6e* mutants^27^, and epiblast-specific deletion of *Ly6e* is compatible with embryonic development until birth, strongly indicating a placental origin of the early embryonic defects.

## Results

### Disrupted labyrinth architecture in *Ly6e*^−/−^ placentae

**Figure 1:**
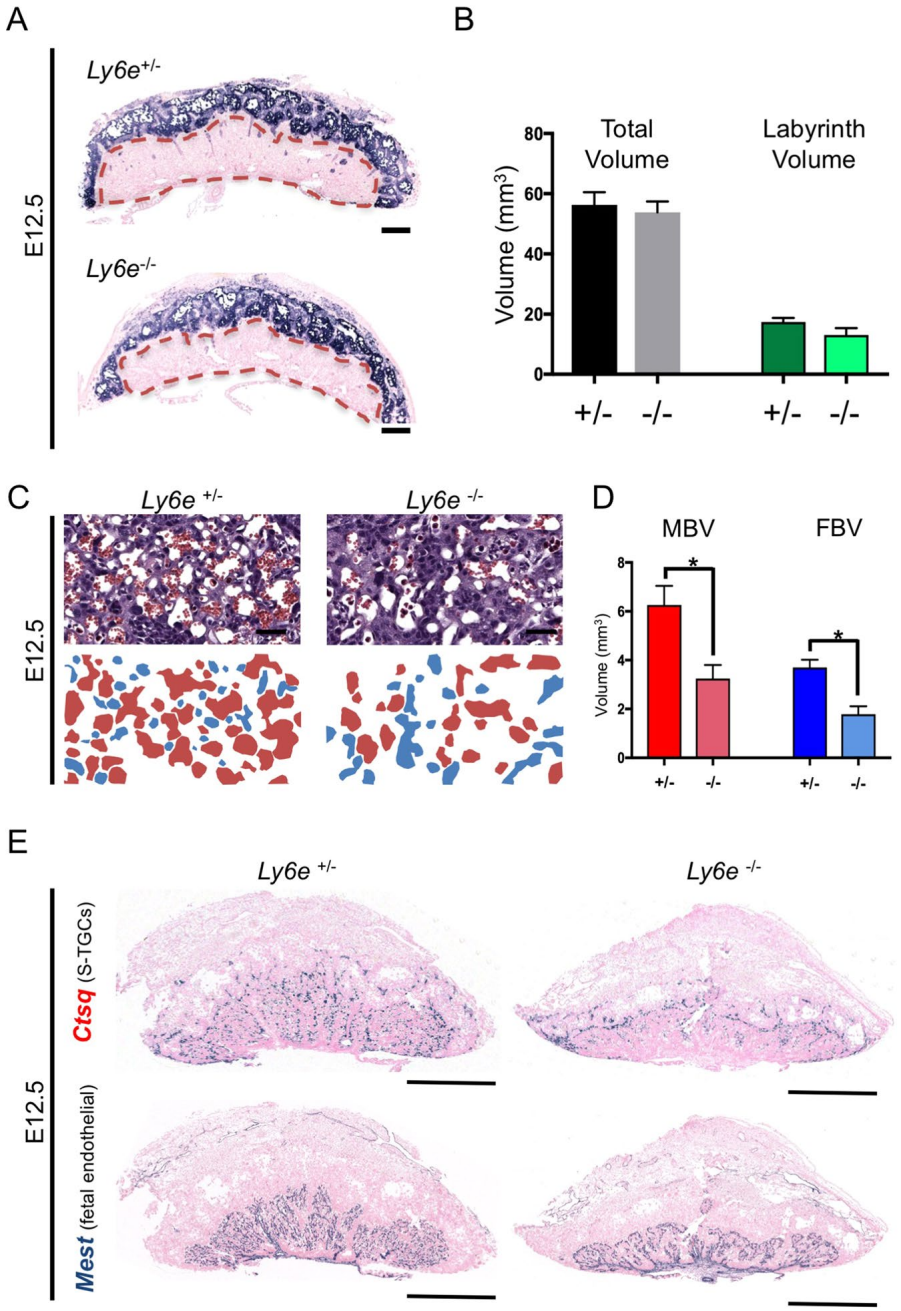
Gross histological assessment of placental morphology and morphometric analysis of the vascular beds in *Ly6e*^−/−^ placentae at E12.5. (**A**) On a gross level, *Ly6e*^−/−^ placentae do not appear dramatically different to wildtype (not shown) or heterozygous controls. *In situ* hybridization using a probe for *Tpbpa* demarcates the spongiotrophoblast layer of the placenta from the decidua above and the labyrinth layer below. The labyrinth layer is outlined by a red dashed line. No apparent differences were seen in *Tpbpa* expression between *Ly6e*^−/−^ and controls. Black scale bar represents 500 μm (**B**) Analysis of *Ly6e*^−/−^ placentae by stereology indicated no differences in total placental volume, nor differences in labyrinth volume (see dashed red lines in panel A). Error bars are +/− s.e.m. (**C**) Analysis of labyrinth organization by stereology in E12.5 *Ly6e*^−/−^ placenta using hematoxylin and eosin staining revealed a disorganization of both the maternal and fetal capillary networks. Red, maternal spaces; blue, fetal spaces. (**D**) Significant decreases in both maternal blood space volume (MBV; red bars) and fetal blood space volume (FBV; blue bars) are apparent in *Ly6e*^−/−^ placentae (**E**) *In situ* hybridisation for *Ctsq* and *Mest* highlight differences in the organisation of maternal blood spaces and fetal blood spaces, respectively. Black bar = 900 μm.

*Ly6e*^−/−^ mutant embryos are morphologically indistinguishable from wildtype littermates ~E13.5, but die by E15.5^27^. As embryonic death can impact placental formation and maintenance^30^, we examined placentae from gestational ages prior to this embryonic lethality (E12.5). We did not observe any qualitative differences in gross or cellular morphology between *Ly6e*^+/+^ or *Ly6e*^+/−^ placentae at either E12.5 or E14.5 (Figs S2,3). Since *Ly6e*^+/−^ embryos develop normally, and are phenotypically normal as adults, we used *Ly6e*^+/−^ placentae as controls for all further comparisons with *Ly6e*^−/−^ placentae. Neither haematoxylin and eosin staining (H&E; data not shown), or *in situ* hybridisation staining for *Tpbpa* (Fig. 1A) revealed any gross morphological differences between *Ly6e* mutant placentae and heterozygous controls at E12.5. Quantification of the overall placental volume and labyrinth volume of mutant placentae did not uncover significant differences with heterozygous controls (Fig. 1B).

The analysis of trophoblast subtype-specific gene expression by *in situ* hybridisation (ISH) allows the examination of placental architecture with greater resolution, and can offer insight into trophoblast specification, differentiation and morphogenesis during gestation^31^. ISH with probes for *Ly6e* confirmed the absence of transcripts in the extraembryonic tissues of mutant samples, and as expected, the heterozygous maternal decidua remained *Ly6e* positive (Fig. S4). Maternal sinusoidal trophoblast giant cells (*Ctsq*+) and fetal endothelial cells (*Mest*+) were readily detectable in E12.5 *Ly6e*^−/−^ placentae, with apparently normal cellular morphology, but their distribution throughout the labyrinth revealed a disorganisation of both circulations (Fig. 1E). Indeed, stereological analysis revealed a reduction in the volume of both the fetal (FBS) and maternal blood spaces (MBS) (Fig. 1D). A shift in the distribution of MBS and FBS luminal sizes within the histological sections (less smaller spaces and more larger spaces; Fig. S5) suggests that branching morphogenesis of both the fetal and maternal blood spaces appears to be impaired by *Ly6e* deletion. Further corroborating this, staining for *Syna*, expressed by syncytiotrophoblast layer I cells^2^, and for *Gcm1*, expressed by syncytiotrophoblast layer II cells^2^, also showed slightly abnormal distribution within the labyrinths of *Ly6e*^−/−^ E12.5 placentae (Figs S6,7), likely reflecting impaired formation of the vascular spaces lined by these cells. ISH probes specific for parietal trophoblast giant cells (*Prl3d*), ectoplacental cone, and later spongiotrophoblast layer cell types (*Prl7b1*, *Prl8a8*) uncovered no discernible differences between *Ly6e*^−/−^ placentae and heterozygous controls (Figs S8–10).

### Increased cell proliferation within the labyrinth of *Ly6e*^−/−^ placentae

**Figure 2:**
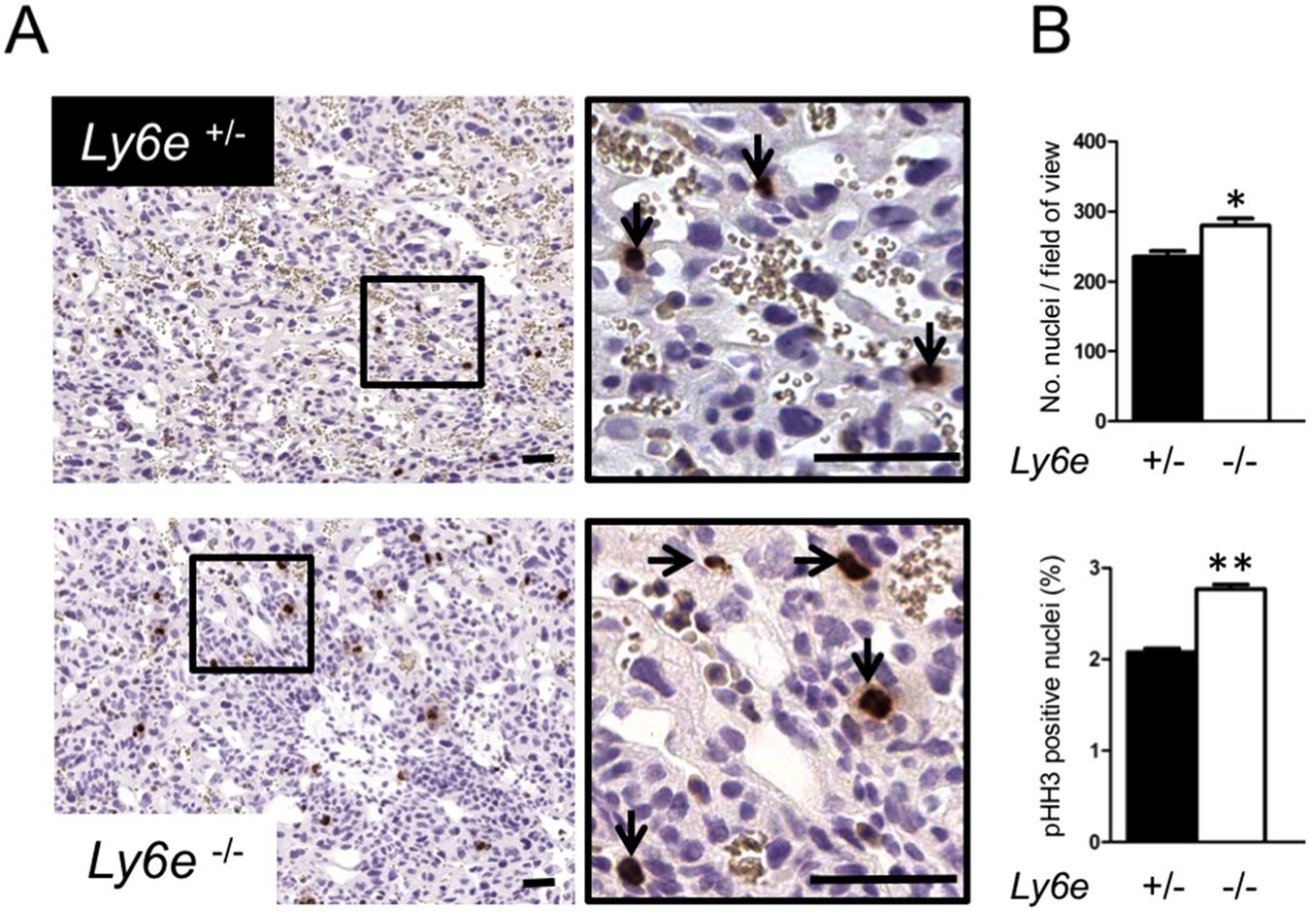
Increased phosphohistone H3 staining in *Ly6e*^−/−^ placentae at E12.5. (**A**) Immunohistochemistry of phosphohistone H3 (pHH3), a mitotic marker, in *Ly6e*^+/−^ and *Ly6e*^−/−^ labyrinth at E12.5. (**B**) Significant increases in the cell density (cell nuclei per field of view) and in the percentage of nuclei that are pHH3 positive were observed in *Ly6e*^−/−^ labyrinth compared with heterozygous controls. Black bar, 50 μm. Error bars are +/− s.e.m.

During labyrinth development, the densely-packed architecture of the chorion gives way to the more porous appearance of the functional placenta as branching morphogenesis produces the tortuous and elaborate networks of fetal and maternal blood vessels lined by thin delicate trophoblast and endothelial cells. Some undifferentiated and proliferative labyrinth trophoblast do persist, expressing trophoblast stem cell genes such as *Eomes*^32^, *Rhox4b*^33^, or *Epcam*^34^, but become restricted to smaller densely packed clusters embedded within the labyrinth. We observed a higher density of these “undifferentiated” trophoblast cell clusters within the labyrinth of *Ly6e*^−/−^ placentae at E12.5 compared with heterozygous controls (Figs 2 and S11). Immunohistochemistry for phosphohistone H3 (pHH3) corroborates this observation as *Ly6e*^−/−^ placentae showed a significant increase in the percentage of pHH3+ cells (Fig. 2). Furthermore, the area positive for *Rhox4b* expression was also increased in *Ly6e*^−/−^ mutants (Fig. S12).

### Disrupted interhaemal membrane formation in *Ly6e*^−/−^ placentae

**Figure 3:**
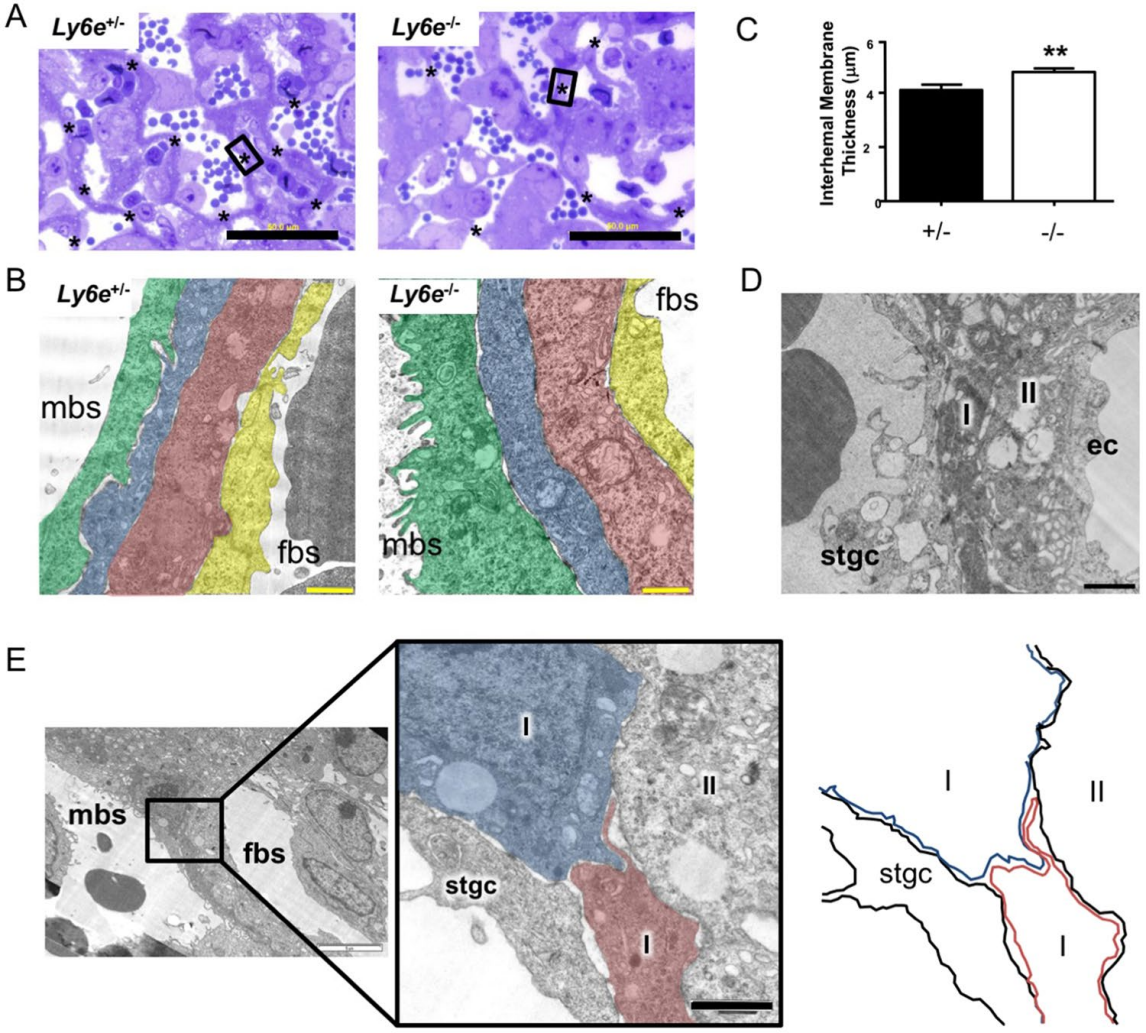
Increased interhaemal membrane thickness in *Ly6e*^−/−^ placentae. (**A**) Areas of extremely thin and highly organised segments of the interhaemal membrane are the sites of placental transport. Toluidine blue stained resin cross-sections (1 μm) of *Ly6e*^+/−^ placentae contained many such segments of the interhaemal membrane (*) separating maternal and fetal blood spaces within the labyrinth, but *Ly6e*^−/−^ placentae contained fewer thin, multicellular segments. (**B**) Pseudo coloured transmission electron micrograph of a characteristically thin transporting segments of the interhaemal membrane in *Ly6e*^+/−^ and *Ly6e*^−/−^ mutant placentae at E12.5. (**C**) A significantly increased thickening (**p < 0.01) of the interhaemal membrane was observed in *Ly6e*^−/−^ mutant placentae compared with heterozygous controls. Black bar, 50 μm; yellow bar, 1 μm; mbs, maternal blood space; fbs, fetal blood space. Pseduocolours: green, sinusoidal giant cells; blue, syncytiotrophoblast layer I; red, syncytiotrophoblast layer II; yellow, fetal endothelial cells. (**D**) Often the cell layers in *Ly6e*^−/−^ interhaemal membranes were observed to have excessive vacuolation and disorganised ultrastructure in layers adjacent to syncytiotrophoblast layer I. Black bar, 1 μm. (**E**) Evidence of a lack of fusion within the syncytiotrophoblast layer I was observed often in *Ly6e*^−/−^ interhaemal membranes, but not heterozygous placentae. Black bar, 1 μm; white bar, 5 μm. Paired littermates (n = 3 pairs) representing the two genotypes were used for all observations, a minimum of three independent labyrinth segments per placenta were examined. Error bars are +/− s.e.m.

On a gross level, the appropriate structure and expansion of the highly-branched architecture of the labyrinth layer is critically important for maximizing the surface area for exchange and for blood flow and perfusion of the placentae. At an ultrastructural, or cellular level, the integrity and organization of the cellular barrier separating the maternal and fetal blood spaces is likewise paramount to the exchange functions of the placenta^35^. Characteristically thin and organized interhaemal membrane structures were less frequently observed in *Ly6e*^−/−^ labyrinths compared with heterozygous controls (Figs 3A; S11), and those that were identified were on average significantly thicker (Fig. 3B,C). Furthermore, *Ly6e*^−/−^ interhaemal membranes had significant ultrastructural abnormalities (Fig. 3B,D); the syncytiotrophoblast layer I was excessively thin and electron dense in some areas (Fig. S13), as if regressing, and failed to undergo fusion altogether in many areas (Fig. 3E). Mutant interhaemal membranes also had visible abnormalities in the layers adjacent to syncytiotrophoblast layer I, such as excessive vacuoles in both syncytial layer II and sinusoidal giant cells (Fig. 3D).

*Lyve1* is expressed in a subpopulation of endothelial cells of the labyrinth, predominantly in endothelium located in the top half of the labyrinth underlying the spongiotrophoblast layer^36^. *Ly6e*^−/−^ placentae have a dramatic 80% decrease in the number of *Lyve1*^+^ cells (Fig. S14).

For a summary of all the morphological and gene expression differences between *Ly6e*^+/−^ and *Ly6e*^−/−^ placentae, see Supplementary Table 2.

### Knockdown of *Ly6e* expression or activation of LY6E alters trophoblast cell-cell fusion and *Syna* expression in TS cell cultures *in vitro*

**Figure 4:**
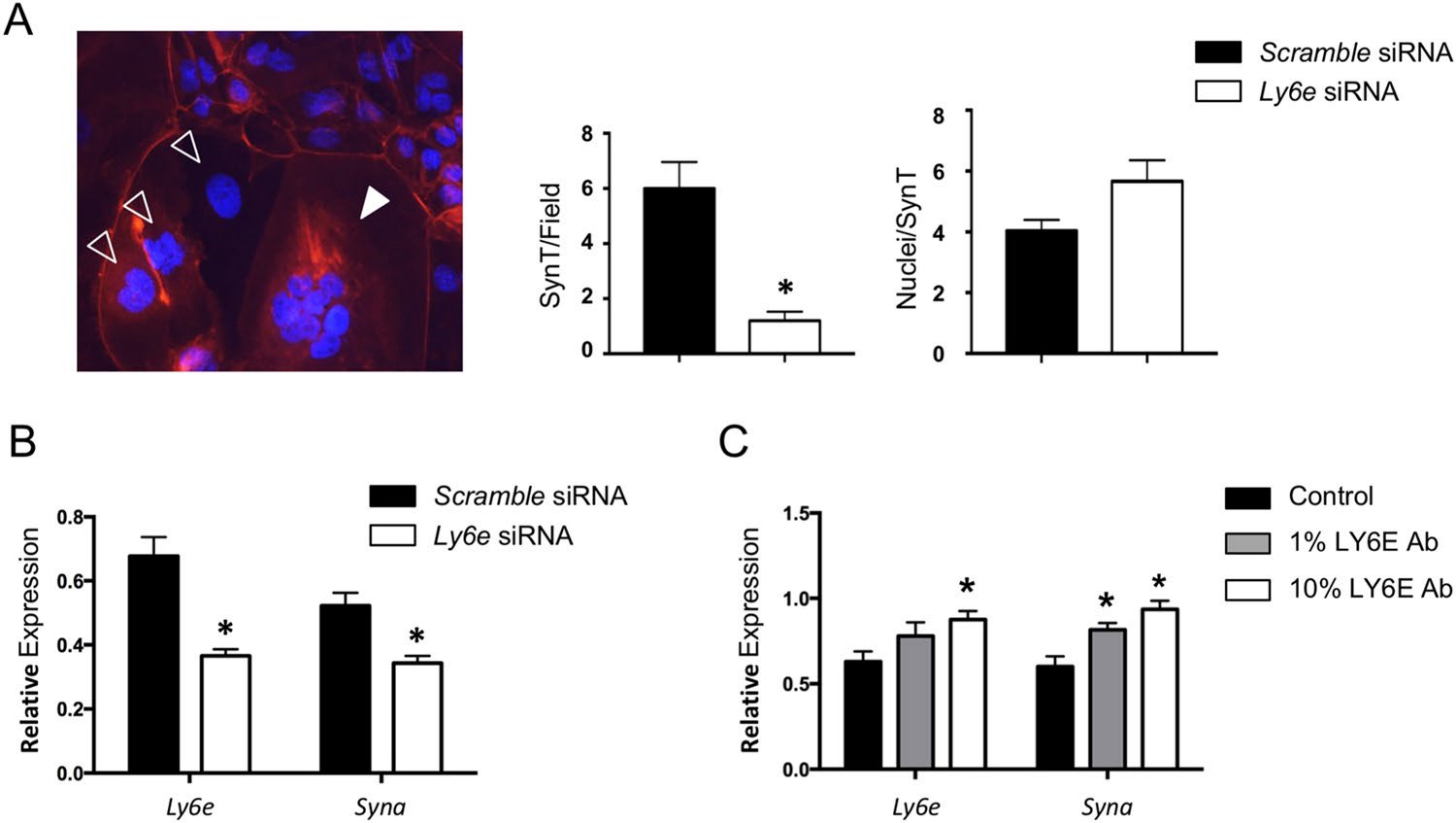
Reduced *Ly6e* expression affects cell fusion and *Syna* expression in differentiating cultured trophoblast cells. (**A**) Mouse TS cells differentiated for 6 days form multinucleated syncytia (multiple DAPI stained nuclei within a single cellular membrane visualised with rhodamine-phalloidin staining). Solid white arrowhead indicates a syncytiotrophoblast cell; Hollow arrow points indicate large, mononuclear trophoblast giant cells. TS cells treated with siRNAs targeted to *Ly6e* formed significantly fewer syncytia upon differentiation than those treated with scramble control siRNAs, however, those that did form contained, on average, the same number of nuclei per syncytia. (**B**) *Ly6e* siRNA treatment resulted in reduced *Ly6e* and *Syna* mRNA expression (*p < 0.05). (**C**) Treatment of TS cells with LY6E antibody has the opposite effect to siRNA treatment, an increase in both *Ly6e* and *Syna* expression (*p < 0.05). PCR data was normalized to *Gapdh* expression. (n = 3).

To confirm a direct role for LY6E in trophoblast syncytia formation, we utilized siRNAs to knockdown (KD) *Ly6e* in differentiating trophoblast stem cells. A reduction in *Ly6e* expression impaired syncytial formation after 6 days of differentiation (Fig. 4A). In differentiated cultures treated with scramble siRNAs, 3.17% of trophoblast (per field of view) were syncytia, while only 0.58% formed syncytia in *Ly6e* siRNA treated cultures. Interestingly, the number of nuclei per SynT cell was not different between scramble and *Ly6e* siRNA treated cultures (Fig. 4A). Consistent with a decrease in syncytia formation, *Syna* expression was also significantly reduced in *Ly6e* KD cells compared with scramble siRNA treated controls (Fig. 4B). *Ly6e* siRNA resulted in ~50% decrease in *Ly6e* mRNA expression levels within the whole of the culture (Fig. 4B).

Previous studies have shown that some antibodies to Ly6 family members such as LY6E and LY6A can stimulate their activities^37–40^. In a complimentary experiment to the siRNA KD of *Ly6e*, we added LY6E antibodies to differentiating TS cell cultures. LY6E antibody treatment resulted in an increase in the expression of *Syna* (Fig. 4C).

### Placental origins of *Ly6e*^−/−^ embryonic lethality; epiblast-specific deletion of *Ly6e* is compatible with embryonic development and adult survival

**Figure 5:**
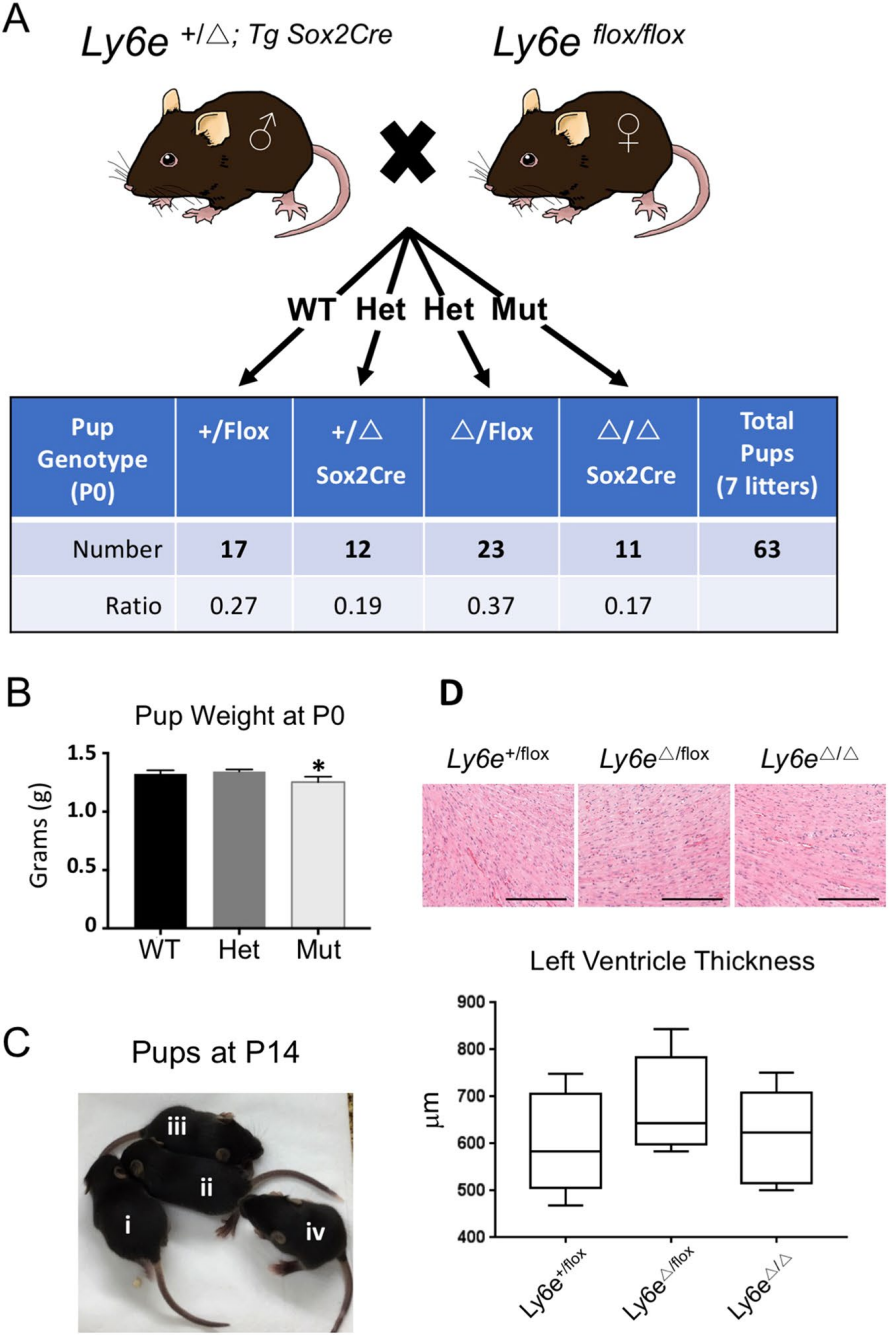
Epiblast-specific deletion of *Ly6e* is compatible embryonic development. (**A**) Crosses with *Ly6e*^+*/Δ; Tg Sox2Cre*^ males with *Ly6e* ^*flox/flox*^ females resulted in the production of viable *Ly6e*^*Δ/Δ*^ embryos at P0. (**B**) *Ly6e*^*Δ/Δ*^ pups we ~5% smaller than wildtype or heterozygote controls at birth (P < 0.05). (**C**) *Ly6e*^*Δ/Δ*^ mice (i and iii) were not significantly different from heterozygous controls (ii and iv) in size, weight, or appearance at P14. (**D**) High magnification of left ventricle myocardium is comparable between genotypes, exhibiting no significant histopathology (6 weeks of age). Scale bar = 100 μm.

Crosses of *Ly6e*^+*/−*^ mice fail to produce any live *Ly6e*^−/−^ embryos at birth^27^, indicating global deletion of *Ly6e* is embryonic lethal. We obtained conditional *Ly6e*^*Flox*^ mice (Zammit DJ, Boyd RL and Classon BJ; unpublished), which contain LoxP sites flanking exons 2 and 4. Deletion of *Ly6e* by Cre Recombinase results in the same loss of coding sequence seen in the original *Ly6e* knockout animals, which replaced exons 2–4 with a PGK-Hygromycin phosphotransferase cassette^27^ (Fig. S15). We initially crossed *Ly6e*^+*/Flox*^ male mice with *Sox2-Cre* female mice^41^ to produce *Ly6e*^+*/Δ*^ offspring also positive for the *Sox2-Cre* cassette. Crosses between *Ly6e*^*+/Δ*^ mice, as observed for *Ly6e*^−/−^ mice, do not produce viable *Ly6e*^*Δ/Δ*^ offspring (see Supplemental Table 3). We then used *Ly6e*^+*/Δ*;*Sox2-Cre*^ males in crosses with *Ly6e*^*Flox/Flox*^ females to produce an epiblast-specific deletion of *Ly6e*, while retaining heterozygous expression of *Ly6e* in the trophoblast of the placenta^41,42^ (Figs 5 and S16). By retaining *Ly6e* expression in the placenta, live, healthy *Ly6e*^*Δ/Δ*^ offspring were born at the expected Mendelian ratios and were indistinguishable from heterozygous or wildtype littermate controls (postnatal (P) day 0) (Fig. 5A–C), with the exception of a small, 5% decrease in body weight. Analysis of the hearts of adult *Ly6e*^*Δ/Δ*^ animals revealed no gross anatomical defects, no significant histopathology to suggest dilated cardiomyopathy, and no evidence of left ventricle wall thinning (Fig. 5D). Adult *Ly6e*^*Δ/Δ*^ animals are also fertile (data not shown). Therefore, the heart defects and embryonic lethality of *Ly6e*^−/−^ embryos most likely have a placental origin, rather than an embryonic requirement for LY6E activity. However, while a placental origin seems probable, it should be noted that without a reciprocal trophoblast-specific deletion of *Ly6e*, we cannot definitively conclude that embryonic lethality in LY6E-deficient embryos is not a consequence of a defect in other extraembryonic tissues, such as primitive endoderm-derived structures like the yolk sac.

## Discussion

Our previous observation that *Ly6e* is expressed in SynT-I cells^20^ prompted us to examine *Ly6e*^−/−^ placentae as a possible explanation for the mid-gestation lethality previously observed in these mice^27^. Indeed, we observed significant placental phenotypes that precede the onset of fetal heart defects and mid-gestation lethality. *Ly6e*^−/−^ placentae appeared grossly normal upon initial collection at E12.5, but closer examination of placentae at the cellular level uncovered a significant disruption to the development, elaboration, and organisation of both the maternal and fetal circulations within the labyrinth. Both MBS and FBS volumes were significantly decreased, and the distribution of cells lining these spaces within the labyrinth, namely *Ctsq*^+^ S-TGCs and *Mest*^+^ endothelial cells, were clearly abnormal. This has been observed in numerous mouse mutant models with labyrinth phenotypes, such as *Lgl2*, *Hoxa13*, *Ncoa1;Ncoa3*, *Cul7*, and *Map2k1;Map2k2* placentae^9,36,43–45^, and is associated with impaired morphogenesis of the placental circulations.

At the time of submission, Bacquin *et al*. reported that LY6E can function as a receptor for the syncytiotrophoblast layer I fusogenic protein Syncytin A^21^. In line with this, we found that both *Ly6e* deletion *in vivo*, and *Ly6e* knockdown *in vitro*, results in syncytiotrophoblast fusion defects. SynT-II cell fusion did not appear affected in *Ly6e*^−/−^ placentas, indicating LY6E plays a cell autonomous role in SynT-I fusion. Furthermore, *Ly6e*^−/−^ mice have very similar placental defects to *Syna* knockout mice^19^, although it should be noted that *Syna*^−/−^ embryos appear to have a slightly more severe phenotype than *Ly6e*^−/−^ embryos. Deletion of *Syna*^19^ results in complete embryonic lethality ~1 day earlier than *Ly6e* mutants^27^, and while we noted a similar increase in densely packed clusters of trophoblast cells within the labyrinth of *Ly6e*^−/−^ placentae, *Syna*^−/−^ placentae appear more severely affected in this regard. Furthermore, we observed rare areas of interhaemal membrane where SynT-I appeared syncytial in nature, although still morphologically abnormal. These discrepancies with the phenotypes observed for *Syna*^−/−^ embryos could result from differences in genetic backgrounds or methodologies. However, less severe phenotypes would also be expected if LY6E were not the sole receptor for SYNA; other cell surface membrane proteins, such as other Ly6 family members, may also have some SYNA binding activity. Nevertheless, the severity of placental and embryonic phenotypes observed in *Ly6e*^−/−^ embryos, and the striking similarity with *Syna*^−/−^ placentae, supports the notion that LY6E likely acts as a main receptor for the fusogen SYNA.

Defects in SynT-I fusion (both in *Syna*^−/−^ and *Ly6e*^−/−^ embryos) appear to have wider impacts on placental morphogenesis. For example, impaired SynT-I fusion is accompanied by a significant increase in the number of diploid trophoblast progenitors within the labyrinth. Interestingly, impaired cytotrophoblast cell-cell fusion, observed in human pregnancies complicated by preeclampsia^46^, is also accompanied by an increase in proliferation of villous cytotrophoblast progenitors^47,48^. However, does the expansion of trophoblast progenitors under these conditions reflect a true increase in proliferation, or rather a build-up of normally proliferating progenitors due to a block in the fusion of progenitors into trophoblast syncytia? Additionally, defects in SynT-I fusion adversely affects the organisation and morphology of adjacent S-TGC and SynT-II cell layers within the interhaemal membrane, resulting in a thicker membrane overall. Gene mutations that increase^13^ or decrease^49^ *Gcm1* expression in SynT-II cells have also been shown to result in disruptions to the architecture of adjacent cell layers and a thickened interhaemal membrane. This is perhaps not surprising considering the interdependency of the individual cell layers in coordinating the morphogenesis of the interhaemal membrane^8^ and mediating vectorial transport at the fetal-maternal interface^50^. Interestingly, reduced *Gcm1* expression in SynT-II resulted in similar changes to the electron density and vacuolation of SynT-I cytoplasm that we observed in *Ly6e* mutants^49^, as did deletion of *Synb*^15^, which impaired SynT-II fusion. This suggests these specific cellular phenotypes may be typical of compromised interhaemal membrane function, rather than direct effects of *Gcm1*, *Synb*, or *Ly6e* deletion. Bainbridge *et al*. proposed that impeded egress of proteins and vesicles through the interhaemal membrane due to defects in one layer might account for the altered morphology of the adjacent layers.

Since disruptions to SynT-II formation via *Gcm1* or *Synb* mutation affected the morphology and ultrastructure of not only the adjacent SynT-I layer, but also the S-TGCs lying an additional cell layer away^49^, we also looked at the endothelial cells of *Ly6e*^−/−^ placentae, which likewise sit two cell layers deep to *Ly6e*-expressing SynT-I cells. We did not observe any ultrastructural abnormalities in fetal endothelial cells from *Ly6e*^−/−^ placentae at the EM level, however, we did observe changes in gene expression. The expression of LYVE1 in wildtype placentae appears to be restricted to a subset of endothelial cells predominantly located at the top of the labyrinth closest to the spongiotrophoblast layer^36^. We observed a dramatic 80% decrease in the number of *Lyve1* expressing cells in the labyrinth of *Ly6e*^−/−^ placentae. While the significance of changes in the proportion of *Lyve1* expressing cells to placental function is currently unclear, what is clear is that disrupted SynT-I formation and fusion has consequences for the whole of the interhaemal membrane, with significant morphological and gene expression changes in all the cell layers.

Two seminal experiments demonstrated that developmental heart defects could be an indirect consequence of impaired placental formation and function: the homozygous deletion of *Pparg*^29^ or *p38a* (*Mapk14*)^28^ resulted in severe cardiac and placental defects, but genetic rescue of the placental defects by tetraploid aggregation rescued the cardiac phenotypes in the null mutant embryos. Thus, a developmental “placenta-heart axis” was proposed. *Ly6e* mutant embryos exhibit cardiac phenotypes and die mid-gestation, but interestingly express negligible *Ly6e* within the developing heart itself, suggesting the cardiac defects may be secondary to an unknown primary placental phenotype^27^. Importantly, when we prevented the placental phenotypes associated with *Ly6e* deletion, by using a *Sox2-Cre* epiblast-specific deletion strategy, homozygous *Ly6e* mutant pups were born and were indistinguishable from littermate controls, including heart morphology. This indicates that the mid-gestation lethality of *Ly6e*^−/−^ embryos previously reported^27^ is most likely the result of placental abnormalities, and not heart defects as originally hypothesised.

The formation and continued elaboration of the maternal and fetal circulations of the placenta represents a critical hurdle for a successful pregnancy, as does the correct organisation and function of the intervening interhaemal membrane. In the current study, we have identified a critical role for LY6E in both these aspects of placental development. Importantly, LY6E is an important mediator of the cell-cell fusion required for syncytiotrophoblast morphogenesis and normal placental function, through its role as a receptor for the fusogenic protein Syncytin A^21^. The cellular phenotypes we observed in the *Ly6e*^−/−^ labyrinth also highlight the interdependence of the layers in development of the interhaemal membrane. Furthermore, the placental phenotypes observed in *Ly6e* mutant embryos precede, and are the most likely cause of, the heart defects and mid-gestation lethality previously described in these mice^27^. Therefore, dysregulation of trophoblast cell-cell fusion, and the resulting defects in placental formation, can be a primary cause of secondary developmental heart defects and embryonic lethality.

## Materials and Methods

### Animals

All procedures involving animals were approved by, and carried out in accordance with, the guidelines of The University of Queensland Animal Ethics Committee. *Ly6e*^+/−^ heterozygous mice^27^, obtained from Prof Richard Boyd (Monash University, Australia) and maintained on a C57BL/6 background, were crossed to obtain *Ly6e*^−/−^ conceptuses. Conditional Ly6e^+/Flox^ animals were also obtained from Prof Richard Boyd (unpublished line) and crossed to *Sox2*-Cre females^41,42^ (purchase from Jackson Labs) to establish *Ly6e*^+/Δ^;*Sox2*-Cre male studs. *Ly6e*^+/Δ^;*Sox2*-Cre males were then crossed with *Ly6e*^Flox/Flox^ females to generate *Ly6e*^Δ/Δ^;*Sox2*-Cre embryos that retain a *Ly6e*^Flox/Δ^;*Sox2*-Cre allele in the trophoblast population of the placenta (see Fig. S2 for further details about the alleles and Fig. S16 about the crosses). Animals were housed under a 12-hour light dark cycle with free access to food and water. Detection of a seminal plug was designated embryonic day (E) 0.5. Embryonic tissue was used for genotyping via PCR analysis (Primers listed in Supplemental Table 1).

### Tissue preparation and histology

For immunohistochemistry and *in situ* hybridization procedures, isolated implantation sites (E8.5-E9.5) or whole dissected placentae (E10.5-E14.5) were fixed overnight at 4 °C in 4% paraformaldehyde (PFA)/1x PBS, processed through an ethanol gradient, cleared in xylene and paraffin embedded. 6–7 μm sections were transferred to Superfrost® Plus slides (Menzel-Gläser) and dried overnight at 37 °C. For ultrathin resin histology, dissected labyrinth layers (E12.5 and E14.5) were cut into 3 × 3 mm cubes with a No. 11 scalpel and fixed overnight at 4 °C in 3% glutaraldehyde in 0.1 M cacodolyate buffer. Subsequent steps were performed in a Biowave (Pelco): after washing with 0.1 M cacodolyate buffer (80 watts for 40 seconds), samples were postfixed with 1% osmium tetroxide (80 watts for 8 minutes), washed in H_2_O (80 watts for 40 seconds under vaccum), dehydrated through a graded series of 50% to 100% acetone (250 watts for 40 seconds each), gradually infiltrated with 1:2, 1:1 and 2:1 epon/acetone (250 watts for 3 minutes each under vacuum) and embedded in 100% epon resin thermally cured at 60 °C for 48hrs. Four labyrinth “cubes” were processed and analysed for each placenta. Semithin (500 nm) and ultrathin (60 nm) sections were cut using glass knives on a Leica EMUC6 microtome. Semithin sections were stained with toluidine blue. Ultrathin sections were contrasted with uranyl acetate and lead citrate and imaged with a JEOL 1010 microscope operated at 80 kV. Experiments were performed at The Centre for Microscopy and Microanalysis at the University of Queensland (Queensland, Australia).

Haematoxylin and Eosin stained placental sections were analysed for placental and vascular space volume. Fetal blood spaces were distinguished by their primitive nucleated erythrocytes. Placental, labyrinth and blood space volumes were calculated using the Cavalieri principle as described previously^35^. ImageScope software (Aperio, CA) was used to obtain images of processed samples. To estimate interhaemal membrane barrier thickness, at least 2 samples of labyrinth for each E12.5 placenta was examined at 20,000× magnification (n = 3 Ly6e^−/+^ and n = 3 Ly6e^−/−^). Interhaemal membranes were imaged at sites representing the thinnest difference between maternal and fetal blood spaces. The width was taken as the average of 3 equally spaced measurements from the region intercepted by the endothelial cell layer and fetal blood space to the region intercepted by the sinusoidal trophoblast giant cell layer and maternal blood space. Measurements were performed using NIH Image J software. For the analysis of left ventricle thickness, transverse H&E sections were measured at 5 locations within the left ventricular wall and averaged. Comparisons were made from sections at equal distances from the apex of the heart, and an n = 3 for each genotype was used. For *Lyve1* positive cell counts, as well as phosphohistone H3 (pHH3) positive and total cell nuclei counts, sections were examined at 400× magnification (min 3 sections from each of n = 3 E12.5 placentae per genotype): 20 fields were acquired randomly from across the labyrinth and both total cell nuclei and positive nuclei/cells were counted (excluding nucleated fetal erythrocytes) using NIH Image J software.

### Immunohistochemistry

For pHH3 immunohistochemistry, paraffin sections were dewaxed, rehydrated and processed for heat-induced antigen retrieval in citrate pH6 buffer (Retriever 2100, Electron Microscopy Sciences). Following washes in tris-buffered saline (TBS), sections were treated to block endogenous peroxidase activity (0.3% H_2_O_2_ in methanol for 20 minutes), and nonspecific binding was limited using 10% goat serum in TBS for 1 hour at room temperature. Sections were incubated in a 1:200 dilution of rabbit polyclonal antibody against pHH3 (Cell Signalling) overnight at 4 °C. Following washes in TBST, sections were incubated with a horseradish peroxidase conjugated goat anti-rabbit antibody (Invitrogen) diluted 1:500 in blocking buffer and detected using diaminobenzidine tablets (Sigma) according to manufacturer’s instructions. Slides were counter-stained with haematoxylin, dehydrated, cleared in xylene and mounted under cytoseal 60 mounting medium (Proscitech, QLD, AUS).

### ***In situ*** hybridization

#### Probes

The plasmids containing gene-specific cDNAs used as templates for the synthesis of sense and antisense DIG-labelled cRNA probes have been previously described; *Gcm1*, *Syna*, *Ctsq*, *Tpbpa*^2^, *Prl3d*, *Prl7b1*, *Prl8a8*^51^, *Rhox4b*^52^, and *Ly6e*^20^. cDNA templates for *Mest* and *Lyve1* were generated by RT-PCR (primers listed in Supplementary Table 1). For plasmids containing gene-specific cDNAs; plasmids were linearized with appropriate restriction enzymes, purified with a Qiagen PCR clean up kit according to manufacturer’s instructions, and used as template for DIG labelled cRNA synthesis with either T7 or SP6 RNA polymerase (10 × DIG labelling mix, Roche). *Ly6e*, *Lyve1* and *Mest* PCR amplicons incorporated T7 and T3 (bold letters in primer sequence) into the reverse and forward primers respectively, and therefore amplicons were used directly as templates for DIG labelled RNA probe synthesis after gel electrophoresis and purification (Qiagen).

#### Hybridization protocol

Hybridizations were carried out as previously described^53^. Slides were imaged using a ScanScope Digital slide scanner (Aperio). Sense probes were used as negative controls to detect for non-specific binding, which was absent for all probe sets used (data not shown).

### Cell Culture

Rs26 trophoblast stem (TS) cells were cultured as previously described^54^. Briefly, TS cells were maintained in an undifferentiated state by culturing in the presence of FGF4 and embryonic fibroblast conditioned medium (CM), and differentiated by withdrawal of FGF4/CM and culturing in TS medium alone. siRNAs directed against *Ly6e* (Qiagen Flextube GS17069 for *Ly6e*; Qiagen, USA) or scramble controls (Qiagen Negative Control siRNA, SI03650325; Qiagen, USA) were transfected into TS cells using Lipofectamine transfection reagent (Life Sciences, USA) at a concentration of 10 μM according to the manufacturer’s instructions. Cells were grown on glass coverslips and allowed to differentiate for 6 days in the presence of siRNAs. Cells were then fixed for 15 minutes at room temperature in 2% PFA, 1x PBS followed by incubation in rhodamine-phalloidin (1:100; Molecular Probes, Invitrogen, USA). Cells were then washed several times in 1x PBS and counterstained in Hoechst 33342 (1:1000; Molecular Probes, Invitrogen, USA) and mounted on slides in Fluorescent Mounting Medium (Dako, USA). N = 4 experimental replicates were performed, with 4–5 random fields of view per replicate, per siRNA treatment counted for both number of syncytia and number of nuclei/syncytia. For antibody treatments, non-purified LY6E antibody (MTS 35; a generous gift from Dr. Richard Boyd, Monash University, USA) was added to TS media during differentiating at concentrations of 1:10 and 1:100 diluted in TS media.

### qPCR

Total RNA was isolated from differentiated TS cell cultures (from 3 independent replicate experiments) using a microspin column-based RNA isolation kit according to the manufacturer’s instructions (RNeasy Kit, Qiagen, USA). 1 μg of total RNA was reverse transcribed using the Quantitect Reverse Transcription Kit (Qiagen, USA). PCR reactions were then prepared using the Quantitect SYBR green PCR kit (Qiagen, USA) according to the manufacturer’s instructions. Primers sequences for *Syna*, *Gcm1* and *Gapdh* are published^33^ and primers for *Ly6e* were purchased from Qiagen (Quantitect Primer Assay QT02280642). Quantitative PCR reactions were conducted in triplicate and *Gapdh* was used as the reference gene. Data was analysed using the Relative Expression Software Tool (REST)^55^ and significance was determined by T-test with p < 0.01.

### Statistical analysis

All comparisons were performed between *Ly6e*^+/−^ and *Ly6e*^−/−^ placentae. Statistical significance (p < 0.05) was determined using 2-tailed Student *t-*test. In the case of *Ly6e*^△/△;TgSox2-Cre^ pup weights, a within blocks ANOVA was used, with each litter considered a block, to account for differences in litter sizes and ages. Results show mean ± s.e.m. Graphs and statistical tests were performed using GraphPad Prism 7 software.

### Data Availability Statement

All data generated or analysed during this study are included in this article (and its Supplementary Information files), or are available from the corresponding author on reasonable request.

### Summary Sentence

Placental *Ly6e* activity is required for normal syncytiotrophoblast formation and placental morphogenesis, and deletion of *Ly6e* leads to mid-gestation embryonic lethality.

## Electronic supplementary material

Supplementary Information
