# Supplementary material for: Deletion of the Syncytin A receptor Ly6e impairs syncytiotrophoblast fusion and placental morphogenesis causing embryonic lethality in mice
Source: Sci Rep. 2018 Mar 2;8:3961. doi: 10.1038/s41598-018-22040-2 (PMC5834536; doi:10.1038/s41598-018-22040-2)
Supplement: Supplementary file 1 — Supplementary Information [file 41598_2018_22040_MOESM1_ESM.pdf]

SUPPLEMENTAL MATERIALS

Title:

Deletion of the Syncytin A receptor Ly6e impairs syncytiotrophoblast fusion and placental morphogenesis causing embryonic lethality in mice.

Authors:

Michael B Langford[1#], Jennifer E Outhwaite[1], Martha Hughes[2], David RC Natale[3], David G Simmons[1]*

1. School of Biomedical Sciences, Faculty of Medicine and Biomedical Sciences, The University of Queensland, St. Lucia, Queensland, Australia.

2. Department of Comparative Biology and Experimental Medicine, Faculty of Veterinary Medicine, University of Calgary, Calgary, AB, Canada.

3. Department of Reproductive Medicine, School of Medicine, University of California, San Diego, California, USA.

# Current Address: The Queensland Brain Institute, The University of Queensland, St. Lucia, Queensland, Australia.

* Corresponding Author

Figure S1

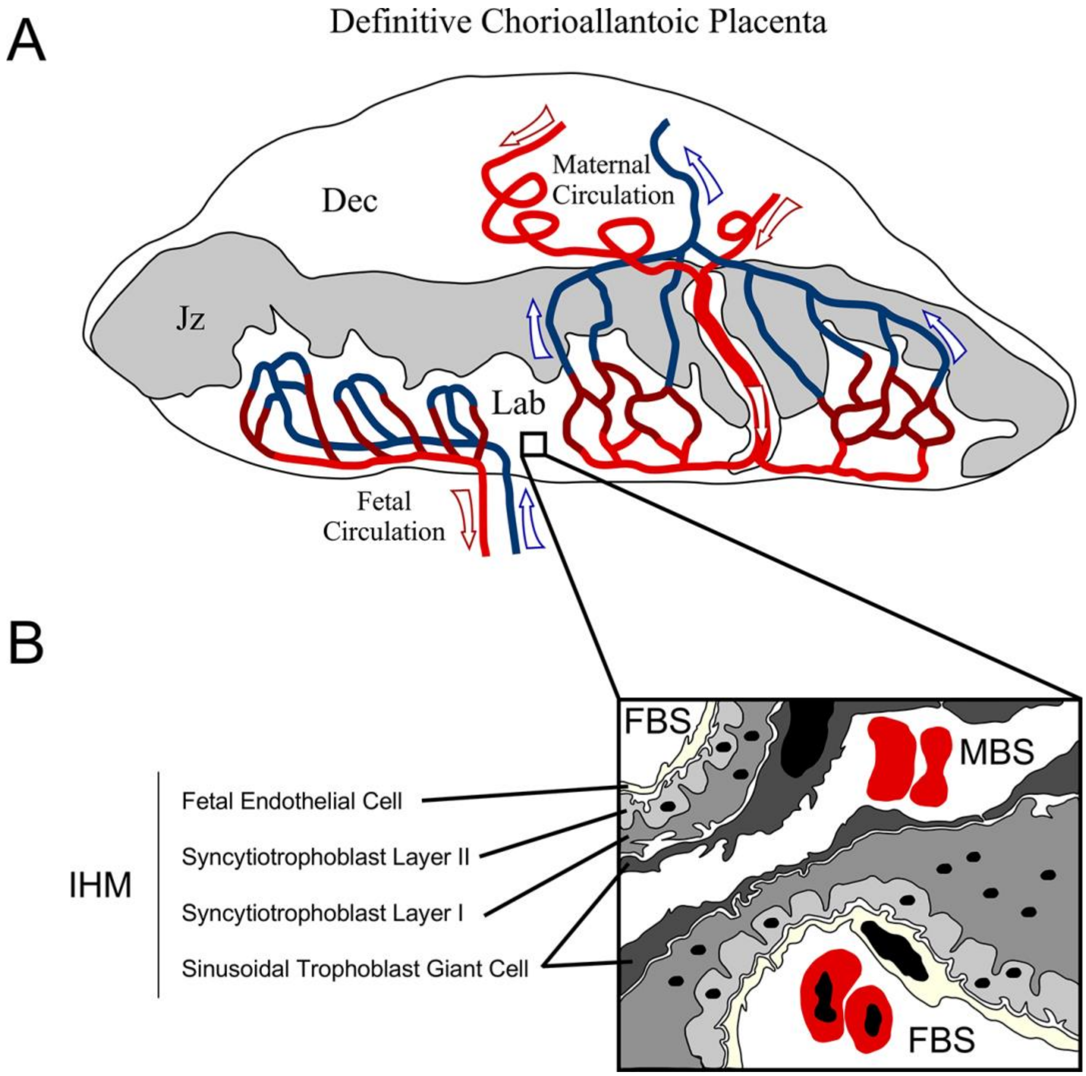

Supplemental Figure 1. Mouse Placental Architecture, Circulations, and Interhemal Membrane. (**A**) The placenta is composed of three "zones" or "layers": the maternal decidua (Dec), an inner "junctional zone" (Jz - made up of parietal trophoblast giant cells, glycogen trophoblast, and spongiotrophoblast), and an inner labyrinth layer (Lab), which is the site of maternal-fetal exchange. Placental circulations (~E12.5-E18.5) are also depicted. Maternal blood enters the placenta through the spiral arteries, which coalesce into ~2-3 central canals that traverse the middle Jz and bring blood to the base of the placental labyrinth layer. Blood then percolates up through a series of tortuous sinusoids until it empties into a venous return system at the Jz-Lab interface where it exits the placenta. Conversely, poorly oxygenated fetal blood enters the base of the labyrinth through larger vessels, flows to the top of the labyrinth layer through a capillary network and back out through the base of the placenta. **(B)** Maternal sinusoids and fetal capillaries are intertwined within the labyrinth, separated by a highly ordered cellular barrier called the interhemal membrane (IHM). The IHM is composed of 4 cell types: fetal endothelial cells that line the fetal vasculature, two intervening layers of syncytiotrophoblast cells, and a layer of sinusoidal trophoblast giant cells that line the maternal sinusoids.

Figure S2

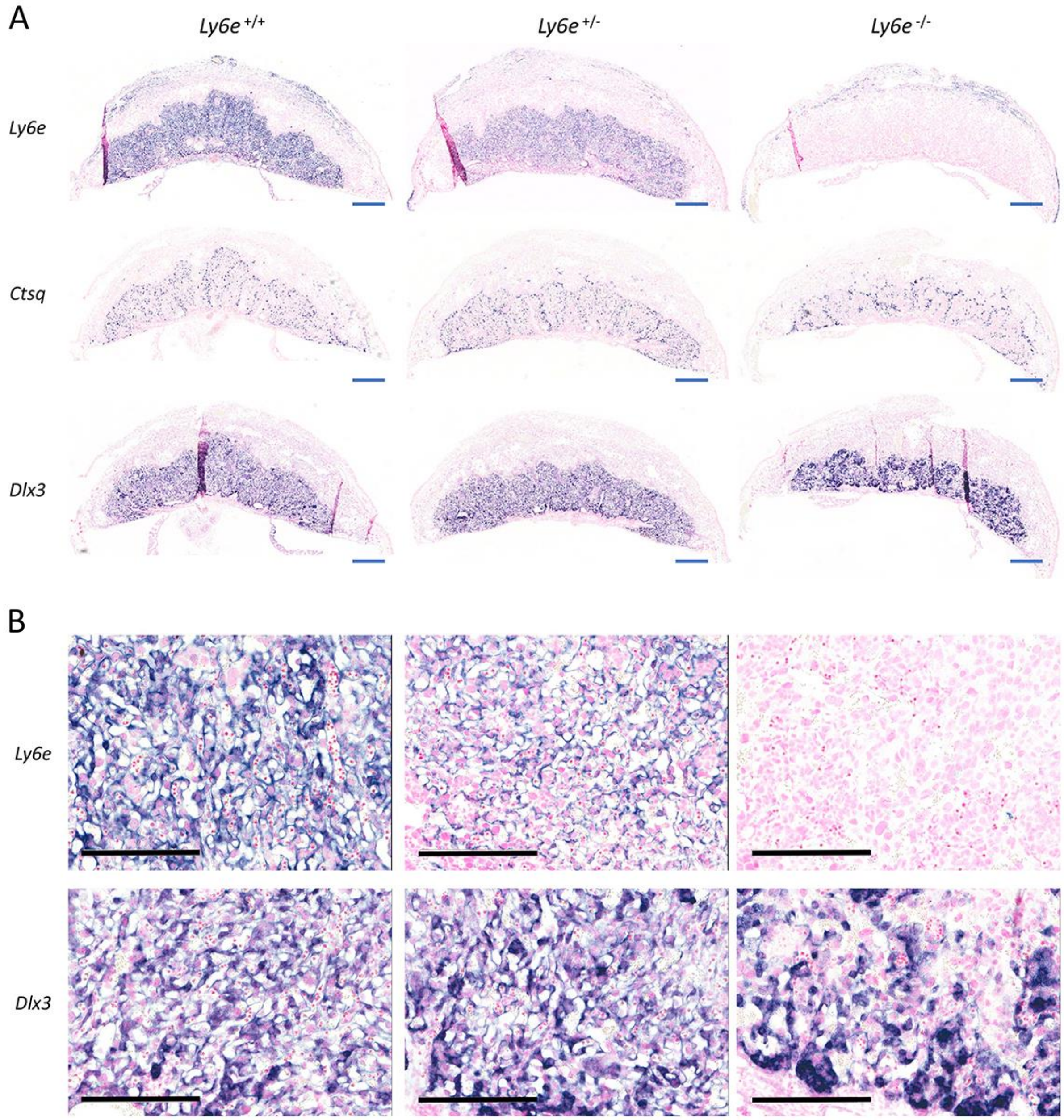

Supplemental Figure S2. Comparison between E12.5 *Ly6e* wildtype, heterozygous and homozygous placentae by in situ hybridization.

(**A**) Low magnification images of *Ly6e* wildtype, heterozygous, and mutant E12.5 placentae – in situ hybridization for *Ly6e, Ctsq* (marker of sinusoidal trophoblast giant cells, S-TGCs, lining maternal blood spaces), and *Dlx3* (a marker of labyrinth trophoblast cells). The distribution of S-TGCs throughout the labyrinth layer looks comparable between wildtype and heterozygous placentae, whereas *Ctsq*$^{+}$ S-TGCs appear abnormally distributed throughout the *Ly6e* mutant placenta. Blue bar = 500 μm. (**B**) High magnification images demonstrate normal distribution of maternal and fetal blood spaces throughout the labyrinth zone of *Ly6e* $^{+/+}$ and *Ly6e* $^{+/-}$ placentae, but abnormal organization of the blood spaces within the labyrinth layer of *Ly6e* $^{-/-}$ placentae. Black bar = 200 μm.

Figure S3

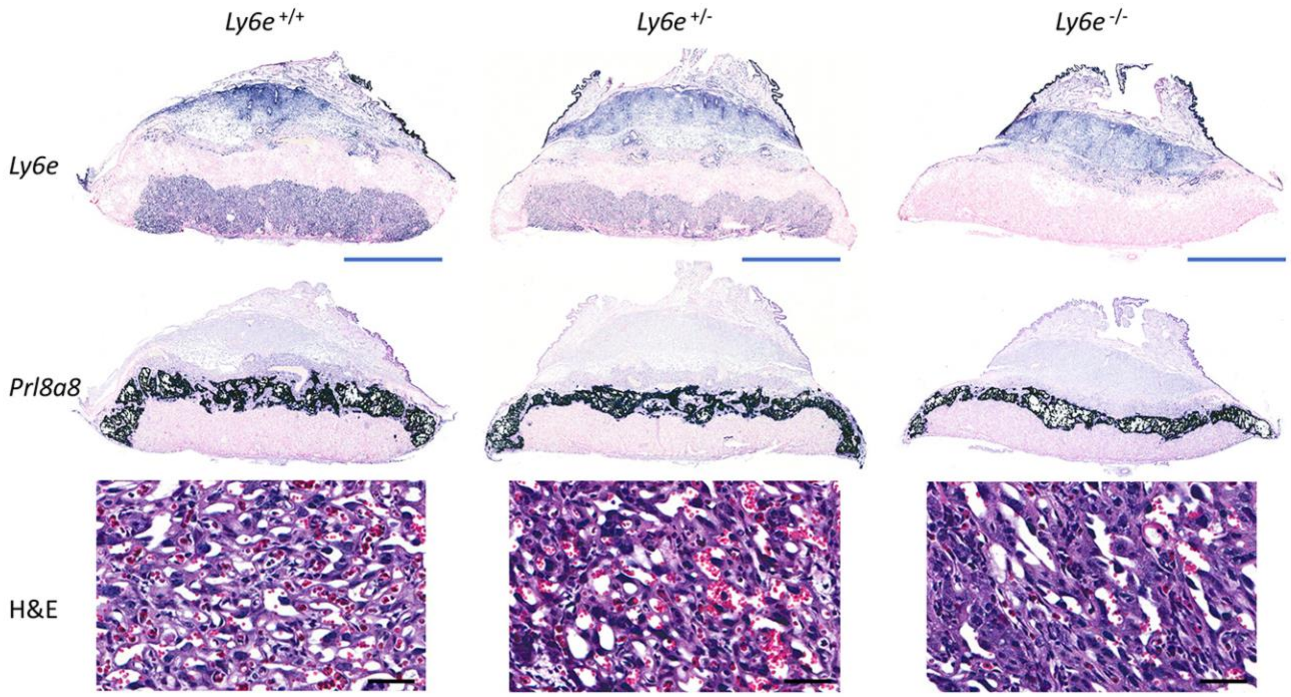

Supplemental Figure S3. Comparison between E14.5 *Ly6e* wildtype, heterozygous and homozygous placentae by in situ hybridization.

Low magnification images of *Ly6e* wildtype, heterozygous, and mutant E14.5 placentae – in situ hybridization for *Ly6e* and *Prl8a8* (marker of junctional zone spongiotrophoblast cells). Note: the labyrinth layer, beneath the *Prl8a8* positive junctional zone, is not different in size between the wildtype and heterozygous *Ly6e* placentae, but *Ly6e* homozygous mutant placentae have a reduced labyrinth thickness by E14.5. Blue bar = 2 mm. High magnification images of H&E sections demonstrate the normal distribution of maternal and fetal blood spaces throughout the labyrinth zones of *Ly6e* wildtype and heterozygous placentae, but abnormal organization of the blood spaces within the labyrinth layer of *Ly6e* mutant placentae. Black bar = 50 μm.

Figure S4.

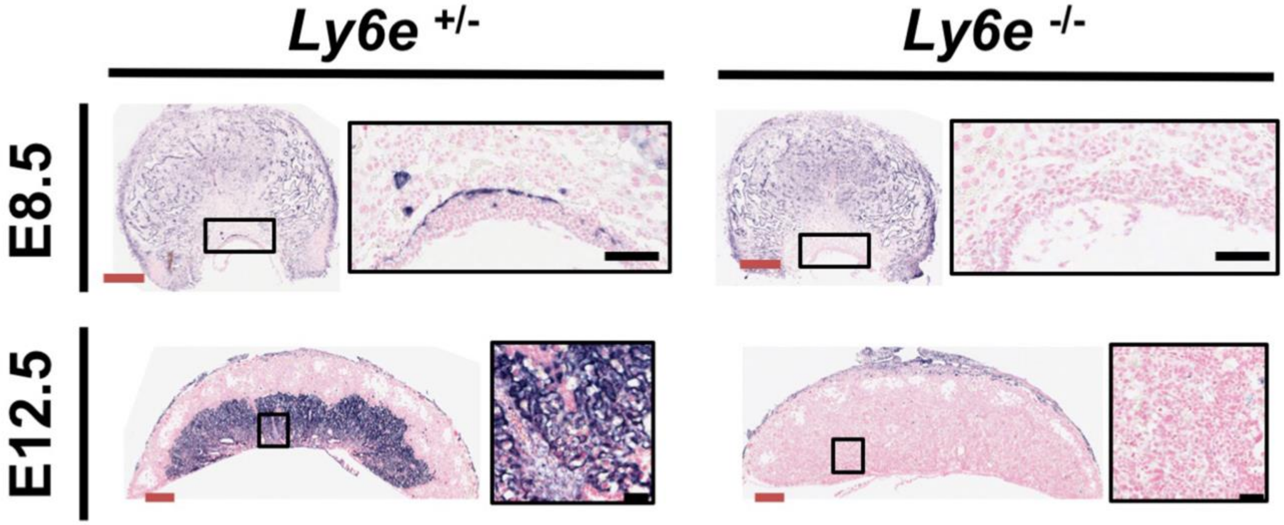

Supplemental Figure 4. In situ hybridization analysis of *Ly6e* in E8.5 and E12.5 *Ly6e*$^{+/-}$ and *Ly6e*$^{-/-}$ placentae.

In situ hybridization confirmed *Ly6e* expression to be absent in *Ly6e*$^{-/-}$ placentae, although positive staining could still be observed in the maternal decidua (which is genetically *Ly6e*$^{+/-}$). Red bar, 500 μm; black bar, 100 μm.

Figure S5

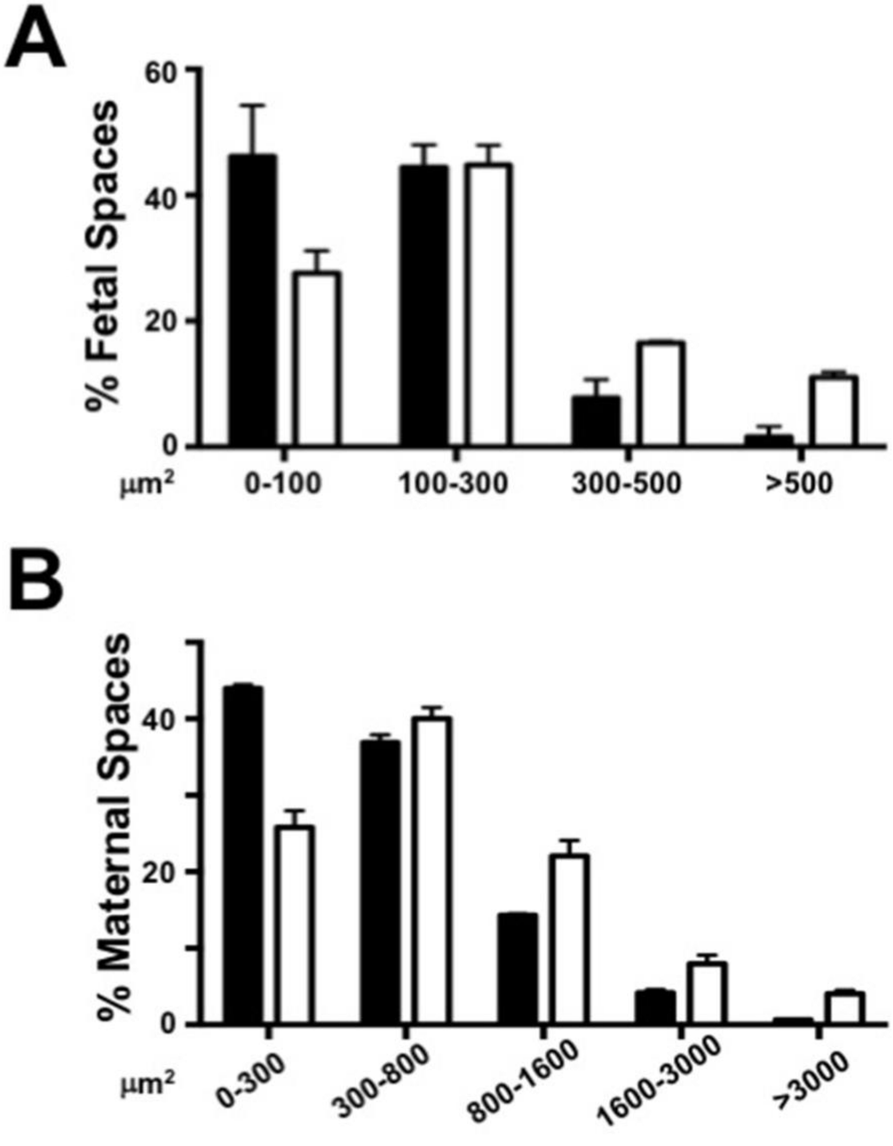

Supplemental Figure S5. Distribution of maternal and foetal blood spaces in E12.5 *Ly6e*$^{+/-}$ and *Ly6e*$^{-/-}$ placentae.

(A) A reduction in the number of 100 µm^2 (or less) sized fetal blood spaces and an increase in larger blood spaces (>300 µm^2) was evident in H&E sections of *Ly6e*$^{-/-}$ placentae. **(B)** A reduction in the number of 300 µm^2 (or less) sized maternal blood spaces and an increase in larger blood spaces (>1600 µm^2) was evident in H&E sections of *Ly6e*$^{-/-}$ placentae. This shift in presence of larger spaces is consistent with impaired branching morphogenesis.

Figure S6

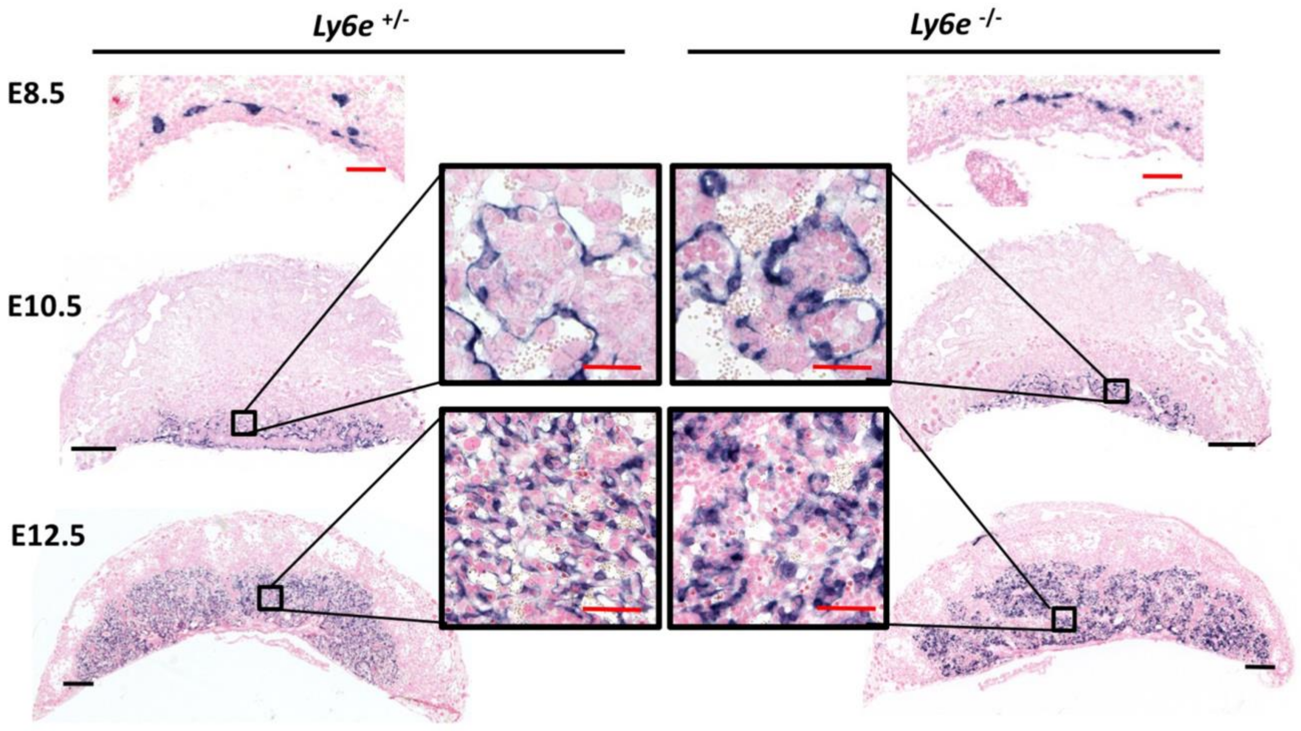

Supplemental Fig 6. In situ hybridization analysis of *Syna* mRNA expression in *Ly6e*$^{+/-}$ and *Ly6e*$^{-/-}$ placentae at E8.5, E10.5, and E12.5.

Syna mRNA expression is evident in the upper half of the chorion at E8.5, and later in the syncytiotrophoblast layer I cells of the E10.5-12.5 placenta in both *Ly6e*$^{+/-}$ and *Ly6e*$^{-/-}$ placentae. Note: the morphology of *Syna*$^{+}$ cells in the *Ly6e*$^{-/-}$ placentae are more rounded, and less characteristically thinned and elongated than in the controls. Furthermore, the distribution of *Syna*$^{+}$ cells within the labyrinth of E10.5-E12.5 *Ly6e*$^{-/-}$ placentae appears disorganised. Black bar, 400 μm; red bar 100 μm.

Figure S7

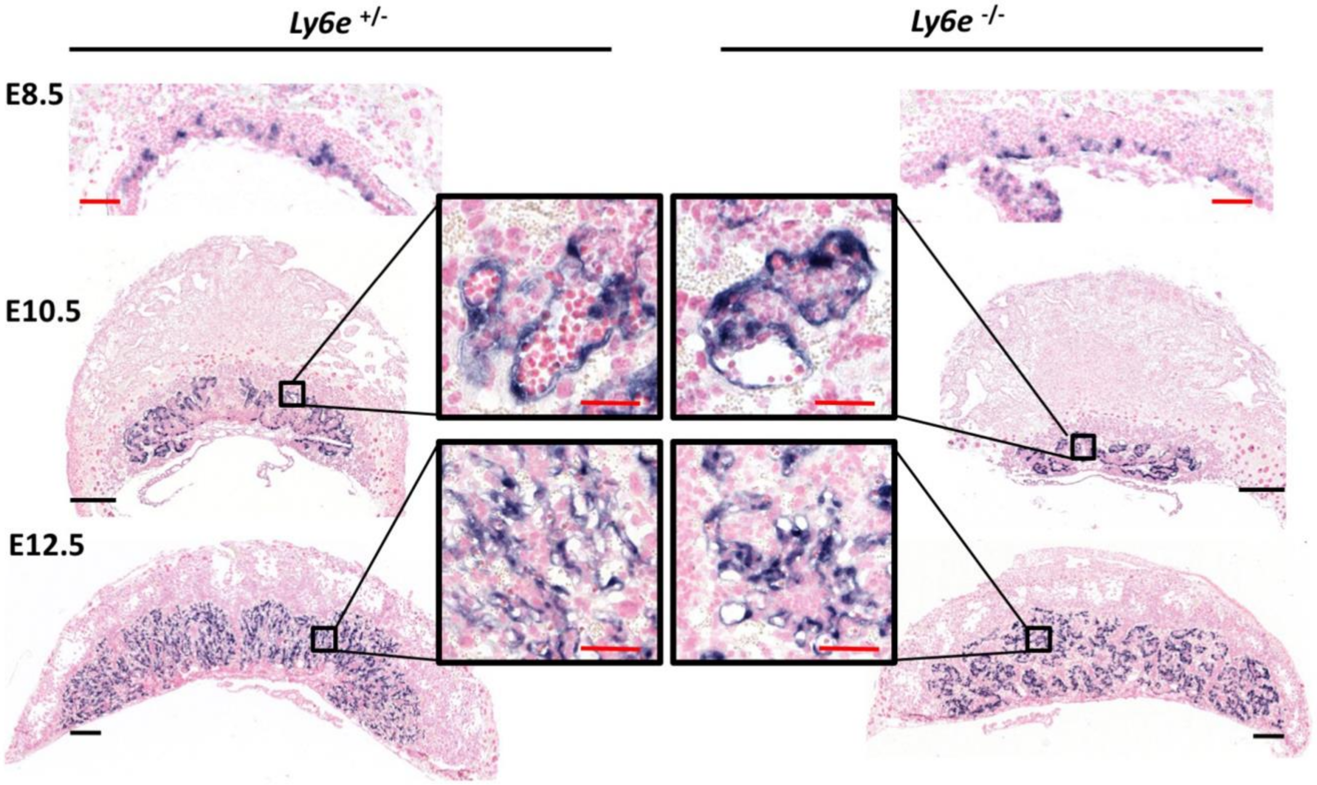

Supplemental Fig 7. In situ hybridization analysis of *Gcm1* mRNA expression in *Ly6e*$^{+/-}$ and *Ly6e*$^{-/-}$ placentae at E8.5, E10.5, and E12.5.

Gcm1 mRNA expression is evident in clusters of cells along the leading edge of the chorion at E8.5, and later in the syncytiotrophoblast layer II cells of the E10.5-12.5 placenta in both *Ly6e*$^{+/-}$ and *Ly6e*$^{-/-}$ placentae. Note: The distribution of *Gcm1*$^{+}$ cells within the labyrinth of E10.5-E12.5 *Ly6e*$^{-/-}$ placentae appears disorganised. Black bar, 400 μm; red bar 100 μm.

Figure S8

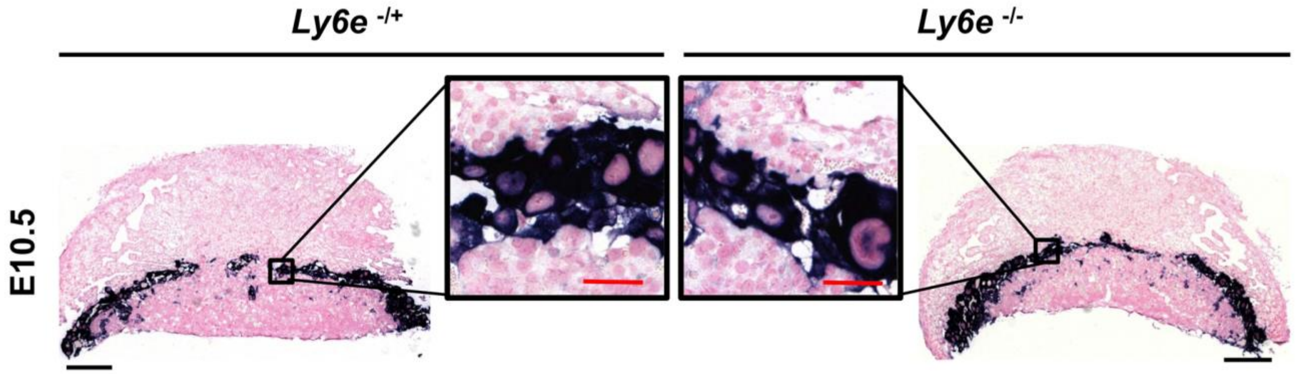

Supplemental Fig 8. In situ hybridization analysis for *Prl3d* (*Pl1*) mRNA expression in *Ly6e*$^{+/-}$ and *Ly6e*$^{-/-}$ placentae at E10.5.

Prl3d positive parietal trophoblast giant cells (P-TGCs) can be clearly seen above the spongiotrophoblast layer in both *Ly6e*$^{+/-}$ and *Ly6e*$^{-/-}$ placentae. No obvious differences in P-TGCs were observed between *Ly6e*$^{+/-}$ and *Ly6e*$^{-/-}$ placentae at any developmental time point examined. Black bar, 400 μm; red bar 100 μm.

Figure S9

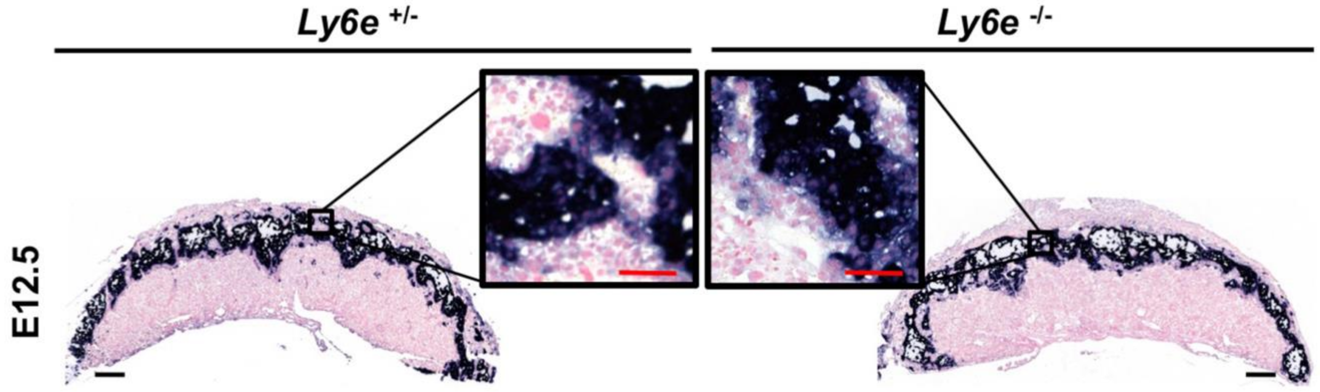

Supplemental Fig 9. In situ hybridization analysis of *Prl8a8* mRNA expression in *Ly6e*$^{+/-}$ and *Ly6e*$^{-/-}$ placentae at E12.5.

Prl8a8 positive spongiotrophoblast cells can be clearly seen within the spongiotrophoblast layer in both *Ly6e*$^{+/-}$ and *Ly6e*$^{-/-}$ placentae (E12.5). No obvious differences in *Prl8a8* staining were observed between *Ly6e*$^{+/-}$ and *Ly6e*$^{-/-}$ placentae at any developmental time point examined. Black bar, 400 μm; red bar 100 μm.

Figure S10

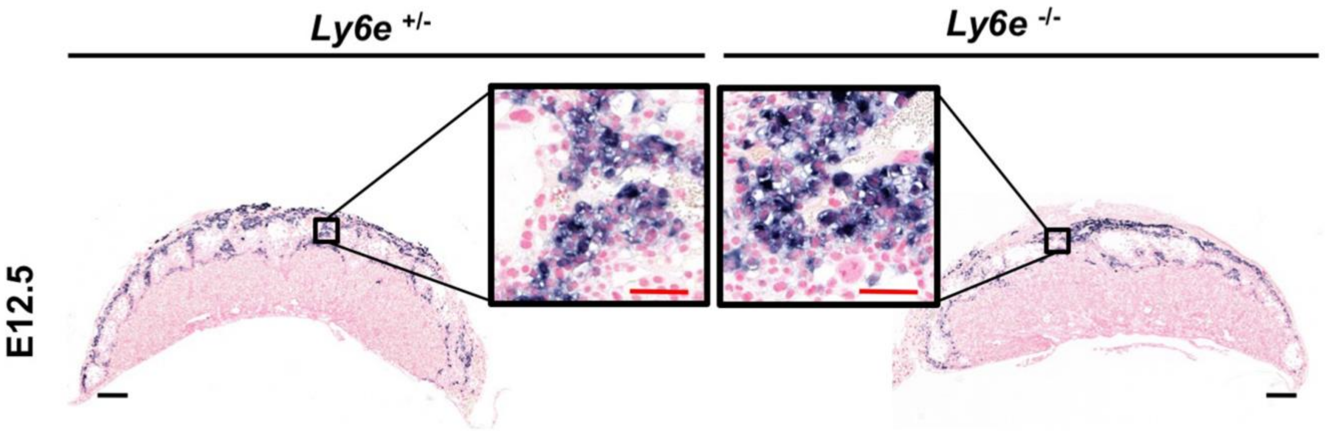

Supplemental Fig 10. In situ hybridization analysis for *Prl7b1* mRNA expression in *Ly6e*$^{+/-}$ and *Ly6e*$^{-/-}$ placentae at E12.5.

Prl7b1 positive glycogen trophoblast cells can be clearly seen within the spongiotrophoblast layer and above the trophoblast giant cell layer within the decidua in both *Ly6e*$^{+/-}$ and *Ly6e*$^{-/-}$ placentae (E12.5). In addition, *Prl7b1* positive spiral artery trophoblast giant cells can also be detected in placentae from both genotypes at E12.5. No obvious differences in *Prl7b1* staining were observed between *Ly6e*$^{+/-}$ and *Ly6e*$^{-/-}$ placentae at any developmental time point examined. Black bar, 400 μm; red bar 100 μm.

Figure S11

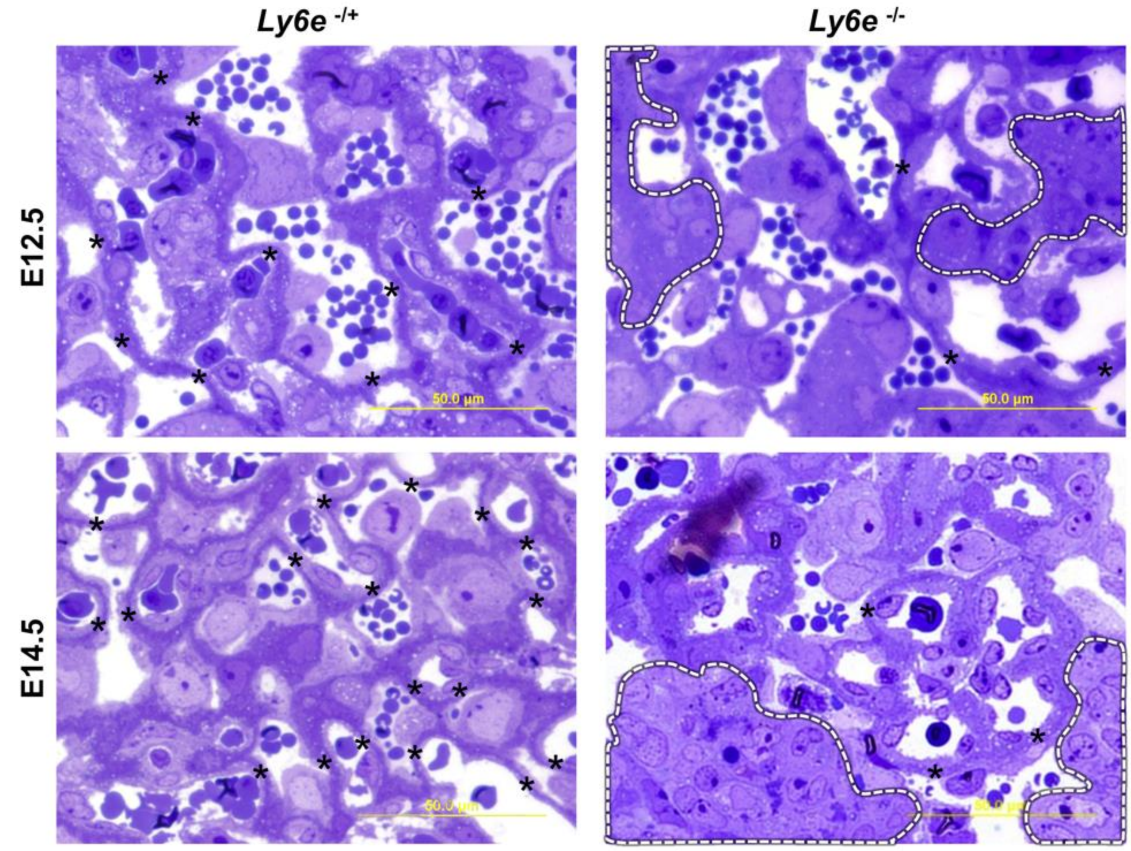

Supplemental Fig 11. Increased number of densely packed clusters of trophoblast cells within the labyrinth layer of *Ly6e*$^{-/-}$ placentae.

Toluidine blue stained resin sections (1 µm) of *Ly6e*$^{-/-}$ placentae at E12.5 (top right) and E14.5 (bottom right) show increased clusters of densely packed trophoblast cells (white hashed lines) within the labyrinth compared with *Ly6e*$^{+/-}$ placentae (top left and bottom left). While clusters of densely packed trophoblast can also be seen occasionally in the labyrinths of *Ly6e*$^{+/-}$ placentae, they are more common in *Ly6e*$^{-/-}$ mutants. * - areas of characteristically thin interhaemal membrane separating endothelial-lined fetal vasculature from trophoblast giant cell-lined maternal sinusoids. Yellow bar, 50 µm.

Figure S12

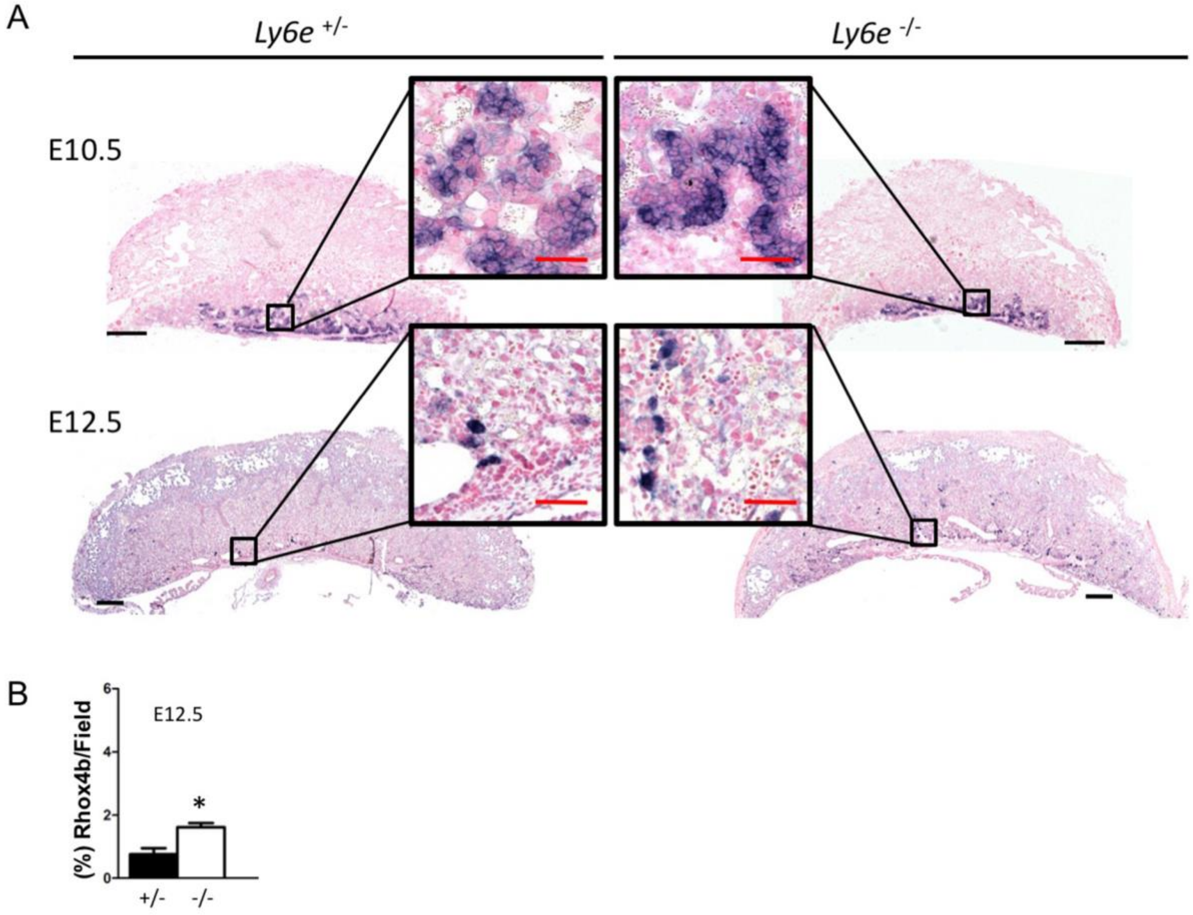

Supplemental Fig 12. In situ hybridization analysis of *Rhox4b* mRNA expression in *Ly6e*$^{+/-}$ and *Ly6e*$^{-/-}$ placentae E10.5, and E12.5.

(**A**) *Rhox4b* mRNA expression is expressed in cuboidal trophoblast cells located predominantly at the base of the developing labyrinth in E10.5-12.5 placenta in both *Ly6e*$^{+/-}$ and *Ly6e*$^{-/-}$ placentae. Note: although the number of *Rhox4b*$^{+}$ cells decreases with gestational age in both *Ly6e*$^{+/-}$ and *Ly6e*$^{-/-}$ placentae, the number of *Rhox4b*$^{+}$ cells in the *Ly6e*$^{-/-}$ placentae is significantly increased compared to age-matched littermate controls. Black bar, 400 µm; red bar 100 µm. (**B**) In E12.5 *Ly6e*$^{-/-}$ placentae, clusters of *Rhox4b*+ cells were distributed more broadly throughout the labyrinth layer, and covered a greater area than in heterozygous or WT littermate controls. Red bar, 100 µm. Error bars are +/- s.e.m.

Figure S13

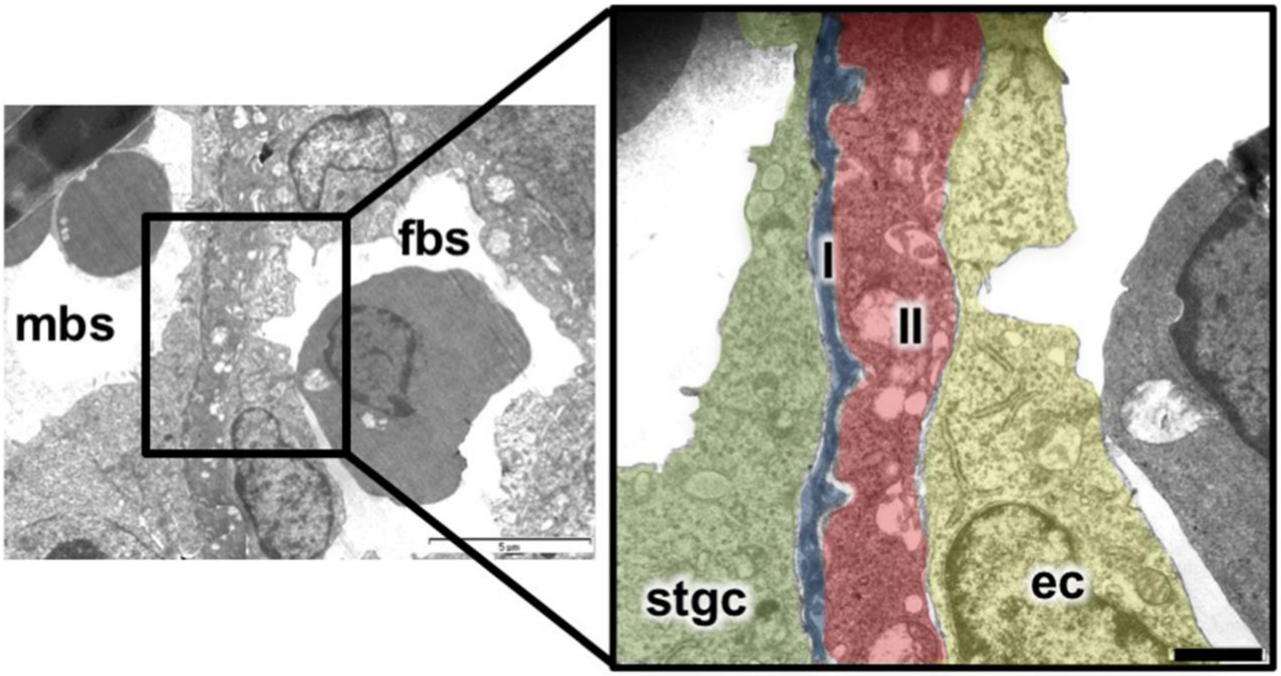

Supplemental Fig 13. Ultrastructural defects in E12.5 *Ly6e*$^{-/-}$ placentae.

Most interhaemal membranes observed in *Ly6e*$^{-/-}$ placentae displayed obvious ultrastructural defects. Some *Ly6e*$^{-/-}$ interheamal membrane segments contained excessively thin and electron dense syncytiotrophoblast layer I, a phenotype never observed in heterozygous labyrinths. Black bar, 1 μm; white bar, 5 μm.

Figure S14

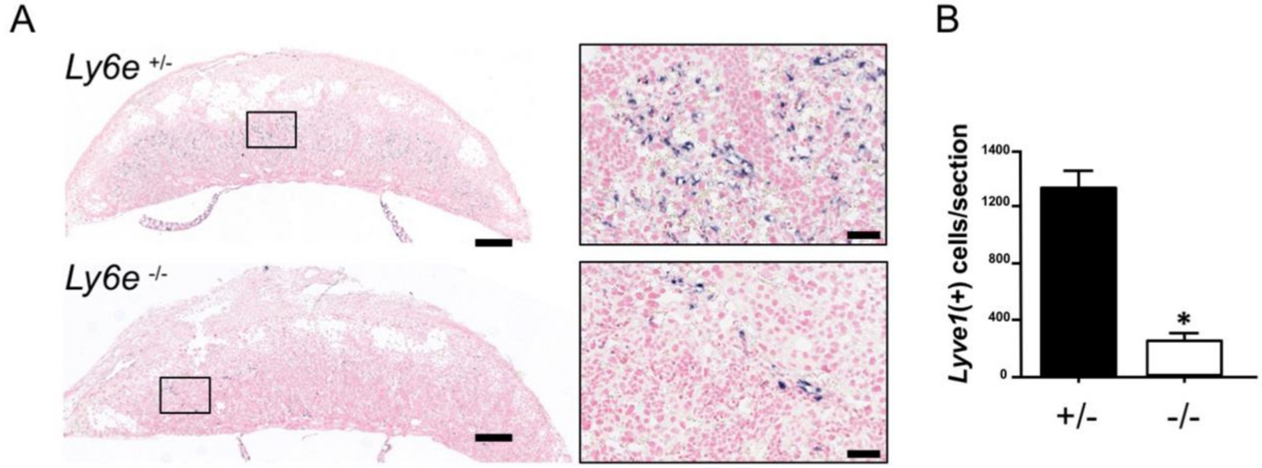

Supplemental Fig 14. Reduced number of *Lyve1* positive cells in E12.5 *Ly6e*$^{-/-}$ placentae.

(**A**) In situ hybridization for *Lyve1* in E12.5 *Ly6e*$^{+/-}$ and *Ly6e*$^{-/-}$ placentae. *Lyve1* expression is restricted to a subset of fetal endothelial cells, predominantly located in the "upper" portion of the labyrinth underlying the spongiotrophoblast layer. E12.5 *Ly6e*$^{-/-}$ placentae contained fewer *Lyve1* positive cells within the labyrinth. (**B**) A significant decrease (*, $p<0.05$) in *Lyve1* cells was observed in *Ly6e*$^{-/-}$ placentae compared with *Ly6e*$^{+/-}$ controls. Black bar – *Ly6e*$^{+/-}$; white bar – *Ly6e*$^{-/-}$. Paired littermates (n=4 pairs) representing each of the two genotypes were used for all measurements; three sections from the midline per placenta were counted. Error bars are +/- s.e.m.

Figure S15

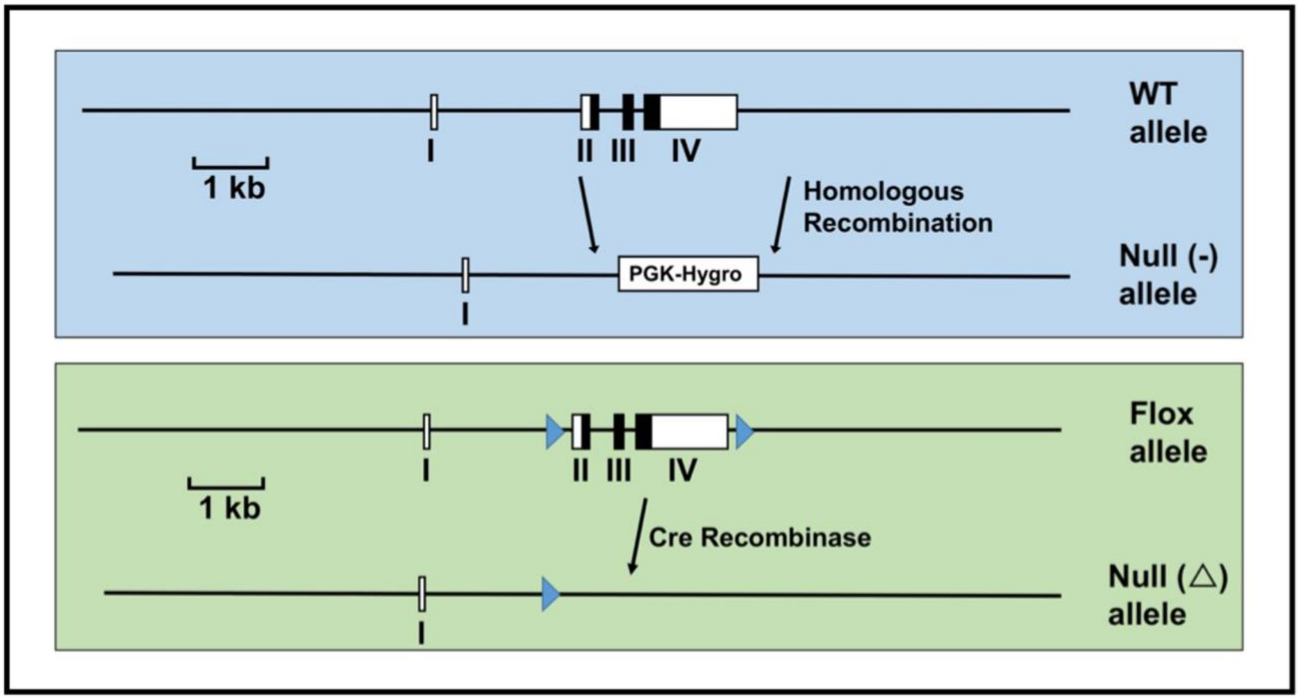

Supplemental Figure 15. *Ly6e* Alleles and conditional crosses.
Null *Ly6e* allele (-) published by Zammit et al.; exons 2-4 replaced with a PGK-Hygromycin cassette. Conditional allele (Flox) contains loxP sites flanking exons 2-4. Once deleted, the null allele (△) has the same genomic structure as the original (-) allele, without the PGK-Hyg cassette.

Figure S16

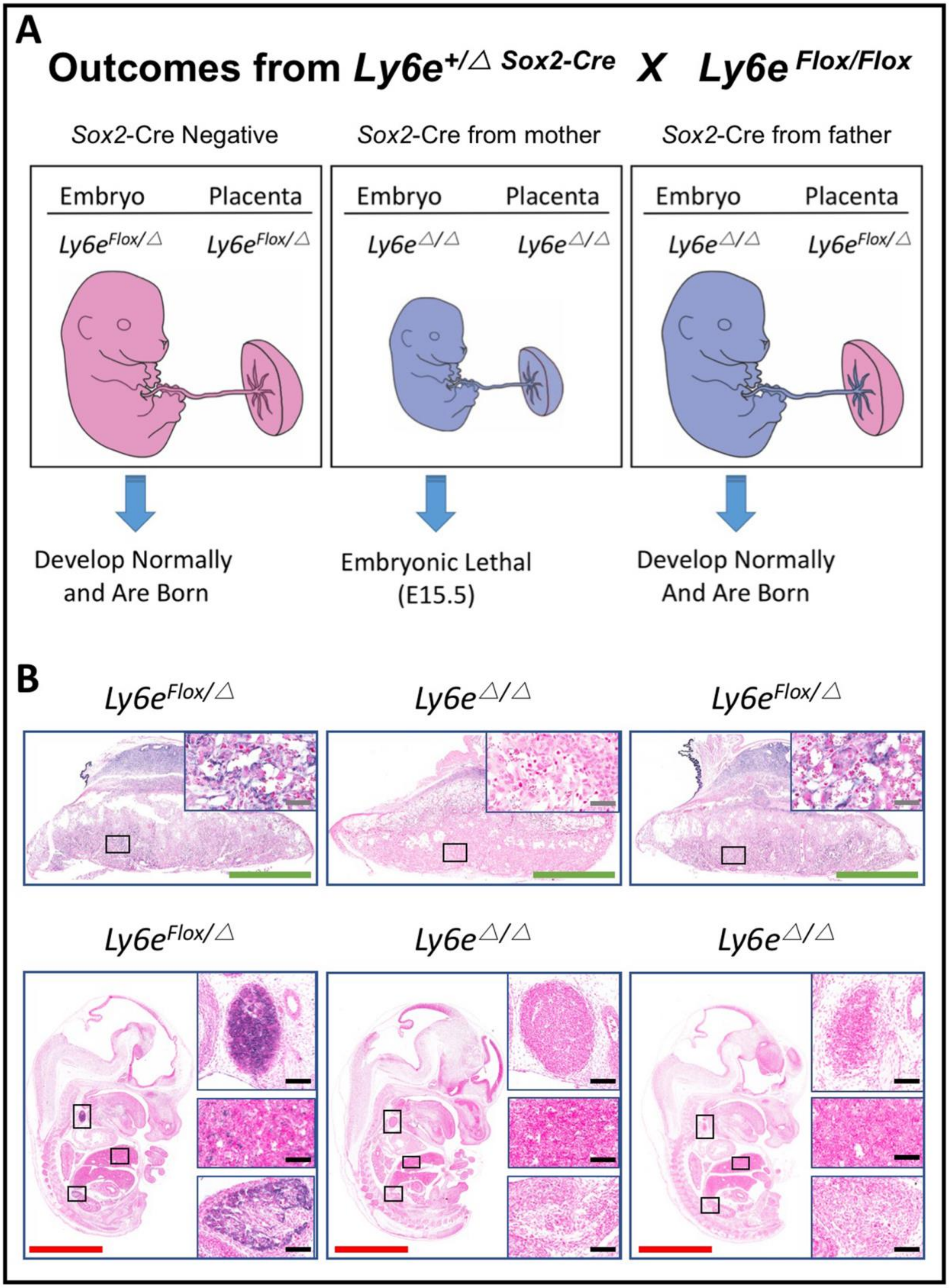
A
Outcomes from Ly6e+/△ Sox2-Cre X Ly6e Flox/Flox
Sox2-Cre Negative
Sox2-Cre from mother
Sox2-Cre from father
Embryo
Placenta
Ly6eFlox/△
Ly6eFlox/△
Embryo
Placenta
Ly6e△/△
Ly6e△/△
Embryo
Placenta
Ly6e△/△
Ly6eFlox/△
Develop Normally and Are Born
Embryonic Lethal (E15.5)
Develop Normally And Are Born
B
Ly6eFlox/△
Ly6e△/△
Ly6eFlox/△
Ly6eFlox/△
Ly6e△/△
Ly6e△/△

Supplemental Figure 16. *Ly6e* conditional crosses.

(**A**) Crosses with *Ly6e*$^{+/\Delta;\ TgSox2\text{-}Cre}$ males and *Ly6e*$^{flox/flox}$ females can produce *Ly6e*$^{\Delta/\Delta;\ TgSox2\text{-}Cre}$ embryos that retain a *Ly6e*$^{\Delta/Flox;\ TgSox2\text{-}Cre}$ placenta, as *Sox2*-Cre is expressed in the epiblast and not expressed in trophoblast of the placenta. Note, crosses with *Ly6e*$^{+/\Delta;\ TgSox2\text{-}Cre}$ females and *Ly6e*$^{flox/flox}$ males result in both the embryo and placenta being *Ly6e*$^{\Delta/\Delta}$ due to *Sox2*-Cre expression in the ovary. Furthermore, crosses between *Ly6e*$^{+/\Delta}$ males and females produced *Ly6e*$^{\Delta/\Delta}$ embryos with the same phenotypes as *Ly6e*$^{-/-}$ embryos (see Supplemental Table 3).

(**B**) In situ hybridization for *Ly6e* in E12.5 placentae and embryos from crosses of *Ly6e*$^{+/\Delta;\ TgSox2\text{-}Cre}$ males with *Ly6e*$^{flox/flox}$ females. Genotypes of placenta/embryos align with those above in section A. Placentas are shown with a higher magnification of the labyrinth layer inset. Embryos are shown with 3 higher magnification insets of tissues that express *Ly6e* in the E12.5 embryo – thymus, liver, and adrenal gland. Note, embryos are missing tails due to sampling for genotyping. Green bar = 2 mm; grey bar = 50 μm; Red bar = 3 mm; Black bar = 200 μm.

Supplemental Table 1

Gene Name	Sequence (5'-3')	RNA Polymerase site
Ly6e forward	**AATTAACCCTCACTAAAGGG**TATCCCACTACTGGGCCTTG3	**T3**
Ly6e reverse	**TAATACGACTCACTATAGGG**TATCGGGGTTGGTCTTTCAG	**T7**
Lyve1 forward	**AATTAACCCTCACTAAAGGG**CATCCCTCGGATTTTCTCAA	**T3**
Lyve1 reverse	**TAATACGACTCACTATAGGG**TCTGCAGGAACTGACAGTGG	**T7**
Mest forward	**AATTAACCCTCACTAAAGGG**GAGAGAGTGGTGGGTCCAAG	**T3**
Mest reverse	**TAATACGACTCACTATAGGG**CGATCACTCGATGGAACCTC	**T7**

Supplemental Table 2

**Comparisons Between *Ly6e*$^{+/-}$ and *Ly6e*$^{-/-}$ Placentae**	
**Gross Morphology (E12.5)**	**Phenotype in *Ly6e*$^{-/-}$ Placentae**
Total Placental Volume	No Difference
Total Jz Volume	No Difference
Total Labyrinth Volume	No Difference
Maternal Blood Volume	**Decreased** in *Ly6e*$^{-/-}$ placentae
Fetal Blood Volume	**Decreased** in *Ly6e*$^{-/-}$ placentae
**EM Morphology of Labyrinth (E12.5)**	**Phenotype in *Ly6e*$^{-/-}$ Placentae**
S-TGC Layer	**Increased** vacuolization and abnormal apical morphology
SynT-I Layer	**Impaired fusion**, abnormal morphology elsewhere, including excessive thinning with increased electron density and increased vacuolization
SynT-II	**Increased** vacuolization
Fetal Endothelial Cells	No Difference
Diploid Trophoblast	**Increased** numbers of diploid trophoblast clusters within the labyrinth
**In Situ Hybridization (E12.5)**	**Phenotype in *Ly6e*$^{-/-}$ Placentae**
Prl3d (*Pl1*)	No Difference (Trophoblast Giant Cell marker)
Prl8a8 (*PlpCγ*)	No Difference (Spongiotrophoblast marker)
Prl7b1 (*PrlpN*)	No Difference (Glycogen Trophoblast marker)
Ctsq	Expressed – but **abnormal distribution throughout labyrinth**. (Sinusoidal Giant Cell marker)
Syna	Expressed (Syncytiotrophoblast Layer I marker)
Gcm1	Expressed (Syncytiotrophoblast Layer II marker)
Mest	Expressed – but **abnormal distribution throughout labyrinth**. (Fetal Endothelial marker)
Rhox4b	**Increased Expression** (Putative Labyrinth Progenitor marker)
Lyve1	**Severely Reduced Expression** (Marker of a Subset of Fetal Endothelial Cells)
**Immunohistochemistry (E12.5)**	**Phenotype in *Ly6e*$^{-/-}$ Placentae**
Phosphohistone H3	**Increased** staining within labyrinth layer

Supplemental Table 3

Viability Data

Ly6e $^{+/-}$ ♂ x *Ly6e* $^{+/-}$ ♀		Embryo/Pup Genotype		
**Age**	**Embryos/Pups (No. Litters)**	*Ly6e* $^{+/+}$	*Ly6e* $^{+/-}$	*Ly6e* $^{-/-}$
E10.5	20 (3)	8	8	4
E12.5	53 (7)	5	37	11
E14.5	90 (12)	34	52	4
E15.5	18 (3)	6	11	1
P14	92 (14)	30	62	0

Ly6e $^{+/\Delta}$ ♂ x *Ly6e* $^{+/\Delta}$ ♀		Pup Genotype		
**Age**	**Embryos/Pups (No. Litters)**	*Ly6e* $^{+/+}$	*Ly6e* $^{+/\Delta}$	*Ly6e* $^{\Delta/\Delta}$
P14	28 (4)	10	18	0

Ly6e $^{Flox/Flox}$ ♂ x *Ly6e* $^{+/\Delta}$ $^{Sox2\text{-}Cre}$ ♀		Pup Genotype			
**Age**	**Embryos/Pups (No. Litters)**	*Ly6e* $^{+/Flox}$	*Ly6e* $^{+/\Delta}$ Sox2-Cre	*Ly6e* $^{\Delta/Flox}$	*Ly6e* $^{\Delta/\Delta}$ Sox2-Cre
P0	12 (3)	5	3	4	0

Ly6e $^{+/\Delta}$ $^{Sox2\text{-}Cre}$ ♂ x *Ly6e* $^{Flox/Flox}$ ♀		Pup Genotype			
**Age**	**Embryos/Pups (No. Litters)**	*Ly6e* $^{+/Flox}$	*Ly6e* $^{+/\Delta}$ Sox2-Cre	*Ly6e* $^{\Delta/Flox}$	*Ly6e* $^{\Delta/\Delta}$ Sox2-Cre
P0	63 (7)	17	12	23	11
